# Effects and mechanisms of supramaximal high-intensity interval training on extrapulmonary manifestations in people with and without chronic obstructive pulmonary disease (COPD-HIIT): study protocol for a multi-centre, randomized controlled trial

**DOI:** 10.1186/s13063-024-08481-3

**Published:** 2024-10-08

**Authors:** Johan Jakobsson, Chris Burtin, Mattias Hedlund, Carl-Johan Boraxbekk, Jonas Westman, Nina Karalija, Per Stål, Thomas Sandström, David Ruttens, Harry R. Gosker, Jana De Brandt, André Nyberg

**Affiliations:** 1 Section of Physiotherapy, Department of Community Medicine and Rehabilitation, Umeå University, Umeå, 901 87 Sweden; 2 REVAL – Rehabilitation Research Center, BIOMED – Biomedical Research Institute, Hasselt University, Diepenbeek, 3590 Belgium; 3 Umeå Centre for Functional Brain Imaging (UFBI), Umeå University, Umeå, 901 87 Sweden; 4 Diagnostic Radiology, Department of Radiation Sciences, Umeå University, Umeå, 901 87 Sweden; 5 Institute of Sports Medicine Copenhagen (ISMC) and Department of Neurology, Copenhagen University Hospital Bispebjerg, Copenhagen, 2400 Denmark; 6 Institute for Clinical Medicine, Faculty of Medical and Health Sciences, University of Copenhagen, Copenhagen, 2200 Denmark; 7 Department of Respiratory Medicine, Ziekenhuis Oost-Limburg, Genk, 3600 Belgium; 8 Faculty of Medicine and Life Sciences, Hasselt University, Diepenbeek, 3590 Belgium; 9 Department of Medical and Translational Biology, Umeå University, Umeå, 901 87 Sweden; 10 Department of Public Health and Clinical Medicine, Umeå University, Umeå, 901 87 Sweden; 11 Department of Respiratory Medicine, NUTRIM School of Nutrition and Translational Research in Metabolism, Maastricht University Medical Center+, Maastricht, the Netherlands

**Keywords:** High-intensity interval training, Pulmonary disease, Chronic obstructive, Randomized controlled trial, Aerobic exercise, Cognitive aspects, Neurodegeneration, Systemic inflammation, Skeletal muscle

## Abstract

**Background:**

Beyond being a pulmonary disease, chronic obstructive pulmonary disease (COPD) presents with extrapulmonary manifestations including reduced cognitive, cardiovascular, and muscle function. While exercise training is the cornerstone in the non-pharmacological treatment of COPD, there is a need for new exercise training methods due to suboptimal adaptations when following traditional exercise guidelines, often applying moderate-intensity continuous training (MICT). In people with COPD, short-duration high-intensity interval training (HIIT) holds the potential to induce a more optimal stimulus for training adaptations while circumventing the ventilatory burden often associated with MICT in people with COPD. We aim to determine the effects of supramaximal HIIT and MICT on extrapulmonary manifestations in people with COPD compared to matched healthy controls.

**Methods:**

COPD-HIIT is a prospective, multi-centre, randomized, controlled trial with blinded assessors and data analysts, employing a parallel-group designed trial. In phase 1, we will investigate the effects and mechanisms of a 12-week intervention of supramaximal HIIT compared to MICT in people with COPD (*n* = 92) and matched healthy controls (*n* = 70). Participants will perform watt-based cycling two to three times weekly. In phase 2, we will determine how exercise training and inflammation impact the trajectories of neurodegeneration, in people with COPD, over 24 months. In addition to the 92 participants with COPD performing HIIT or MICT, a usual care group (*n* = 46) is included in phase 2. In both phases, the primary outcomes are a change from baseline in cognitive function, cardiorespiratory fitness, and muscle power. Key secondary outcomes include change from baseline exercise tolerance, brain structure, and function measured by MRI, neuroinflammation measured by PET/CT, systemic inflammation, and intramuscular adaptations. Feasibility of the interventions will be comprehensively investigated.

**Discussion:**

The COPD-HIIT trial will determine the effects of supramaximal HIIT compared to MICT in people with COPD and healthy controls. We will provide evidence for a novel exercise modality that might overcome the barriers associated with MICT in people with COPD. We will also shed light on the impact of exercise at different intensities to reduce neurodegeneration. The goal of the COPD-HIIT trial is to improve the treatment of extrapulmonary manifestations of the disease.

**Trial registration:**

Clinicaltrials.gov: NCT06068322. Prospectively registered on 2023-09-28.

**Supplementary Information:**

The online version contains supplementary material available at 10.1186/s13063-024-08481-3.

## Introduction

### Background and rationale

Chronic obstructive pulmonary disease (COPD) is currently the third leading cause of death [1], affecting 400 million individuals globally with prevalence rates still climbing [2, 3]. Today, COPD is recognized as a multifactorial systemic disease and not only a respiratory disease, given its association with numerous extrapulmonary manifestations such as decreased muscle [4], brain [5], and cardiovascular function [6]. Regardless of lung impairment severity, these manifestations significantly influence clinical outcomes, including exercise capacity, dyspnoea, quality of life [4], daily life activity [7], healthcare service utilization, and mortality [4, 8]. For instance, the coexistence of COPD and cognitive dysfunction has an additive effect on all-cause hospitalization and a threefold increase in all-cause mortality [8], while quadriceps muscle atrophy is associated with a fourfold increase in mortality [4].

Exercise training is the cornerstone in the non-pharmacological treatment of COPD [2, 9] as it enables increased cardiorespiratory fitness, muscle function, and quality of life, while also reducing dyspnoea, ventilatory demands, and mood disturbances [9]. Nevertheless, adherence to traditional exercise guidelines returns inadequate responses in up to half of all individuals with COPD [10]. This is often attributable to the ventilatory constraints during prolonged exercise that causes many individuals with COPD to cease exercise before their cardiovascular system or skeletal muscles undergo sufficient stress to attain significant adaptation [11]. Thus, there is an urgent need for innovative exercise methods.

Research in healthy individuals has demonstrated that high-intensity interval training (HIIT) is often superior to moderate-intensity continuous training (MICT) in enhancing cardiorespiratory fitness, cognitive function, and muscle function [12, 13]. In individuals with COPD, HIIT holds the potential to address the limitations imposed by continuous training through the inclusion of repeated bouts at significantly higher external intensities than can be sustained during MICT. This, in turn, allows for prolonged exposure of peripheral muscles to high-intensity exercise [14]. However, the feasibility and effects of different HIIT modalities in COPD remain underexplored.

A novel concept of controlled supramaximal HIIT involves 10 × 6 s cycling intervals [15, 16] at intensities above maximal aerobic capacity, that is, higher than the external intensity that results in maximal oxygen uptake during a cardiopulmonary exercise test (CPET). Importantly, while the intensity is supramaximal, it is dosed and performed at a given fraction of the maximum capacity that the person can produce during the bout (6 s). In contrast to an all-out regimen, or sprint interval training (SIT), this allows for the introduction and titration of intensity to the highest acceptable level without the individual feeling unwell during the process.

The concept of short-duration intervals could make supramaximal HIIT specifically suitable for people with COPD, as it lowers the demand on the ventilatory system while enabling a high-exercise intensity. Preliminary data from our research group reveal that supramaximal HIIT enables more than a threefold increase in exercise intensity compared to MICT, with 85% of individuals with COPD preferring supramaximal HIIT over MICT [17]. This form of HIIT has recently demonstrated a larger domain-specific effect on working memory and muscle function compared to lower-intensity exercises in healthy older adults [16]. However, rigorous long-term studies investigating the feasibility and chronic effects of supramaximal HIIT in people with COPD are currently lacking.

Prior research on HIIT in people with COPD has shown that HIIT yields comparable benefits to MICT in regard to cardiorespiratory fitness and exercise capacity [14]. However, these studies have been utilizing intervals lasting from 30 s up to 4 min, necessitating intensities close to or below maximal aerobic power (MAP; around 80–90% of MAP) obtained during a maximal CPET. In contrast, supramaximal HIIT seems to enable exercise intensities higher than 200% of MAP [17].

Enabling exercise at high intensities is important since intensity is the key factor for activating adenosine monophosphate-activated protein kinase (AMPK) and its downstream target, peroxisome proliferator-activated receptor gamma coactivator (PGC)-1α, which is a key regulator of mitochondrial biogenesis in human skeletal muscle [18–20]. For example, hypomethylation of promotor regions of PGC-1α after acute high-intensity exercise [21] and an intensity-dependent increase in mRNA expression of PGC-1α after acute exercise are observed [18]. In individuals with COPD, PGC-1α expression in skeletal muscles is reduced, suggesting a lower drive for mitochondrial biogenesis, which in turn reduces oxidative capacity and limb muscle function [4]. Moreover, decreased PGC-1α signalling was associated with lower expression of kynurenine aminotransferase (KAT), indicative of less kynurenine clearance in the muscle in people with COPD [22]. Consequently, more circulating kynurenine can pass the blood–brain barrier and in the brain kynurenine metabolites (e.g., kynurenic acid) may negatively affect brain health. Additionally, the activation of the AMPK-PGC-1α pathway is vital for stimulating the production of brain-derived neurotrophic factor (BDNF) in the brain [23]. BDNF is essential to the development and maintenance of the central nervous system, encompassing brain and cognitive function [24]. Increased BDNF levels promote neurogenesis, synaptic plasticity, and overall cognitive function [25]. Interestingly, HIIT modalities are recommended for promoting brain health and elevating BDNF levels [26].

In COPD, research on brain health and cognitive function is limited [27, 28], and the pathogenesis of cognitive dysfunction and neurodegeneration is not fully understood [28, 29]. In healthy individuals, it appears that oxidative stress, lack of physical activity, and increased inflammation can accelerate the ageing process, thereby exacerbating age-related neurodegenerative changes [30]. In COPD, pro-inflammatory markers such as C-reactive protein (CRP), activated leukocytes, interleukin 6 (IL-6), IL-8, tumour necrosis factor-alpha (TNF-α), and fibrinogen are closely linked with disease development [31]. Recent studies have posited that systemic inflammation plays an essential role in inducing cognitive dysfunction and brain structural changes in COPD, warranting further attention [28, 29, 32–35].

In this regard, the lack of longitudinal studies is a significant limitation. They are a prerequisite [36] to elucidate the mechanisms underlying cognitive dysfunction in people with COPD, such as the potential acceleration of neurodegenerative changes resulting from increased systemic inflammation [31], as proposed in healthy adults [30]. As acknowledged in recent reviews [27, 29, 37], research on exercise as a potential treatment of cognitive dysfunction in people with COPD is still in its infancy. For instance, no trial has compared the effect of different exercise intensities on cognitive function in people with COPD [27, 29, 37]. Moreover, it remains unknown whether exercise can influence the trajectories of neurodegeneration over time.

Considering the current research gaps, the COPD-HIIT trial aims to investigate the effects and mechanisms of 12 weeks supramaximal HIIT compared to MICT on stationary bicycles in people with COPD and matched healthy controls, focusing on clinical outcomes including brain health, cardiorespiratory fitness, and muscle function (phase 1). Further, we intend to examine the effects of 24 months of exercise training (supramaximal HIIT or MICT) compared to usual care in people with COPD on the trajectories of neurodegeneration and assess the extent to which inflammation and exercise intensity impact neurodegeneration and exercise responses over time (phase 2). Through the COPD-HIIT trial, we aim to lay the foundation for a ground-breaking approach to manage extrapulmonary manifestations in people with COPD and provide evidence delineating how to improve several important health factors in this disease. Additionally, we strive to explore if the effects and mechanisms from exercise training are similar, or how they differ, between people with COPD and matched healthy controls.

### Objectives

#### Primary objectives

The primary objectives are as follows:

- To determine and compare the effect of 12 weeks of supramaximal HIIT and MICT on cognitive function, cardiorespiratory fitness, and quadriceps power in people with COPD compared to matched healthy controls (phase 1);
- To determine and compare the effect of 24 months of supramaximal HIIT, MICT, and usual care on cognitive function, cardiorespiratory fitness, and quadriceps power in people with COPD (phase 2).

#### Secondary objectives

The secondary objectives include the determination of the effects of supramaximal HIIT, MICT, and usual care on:

- Exercise tolerance and anaerobic exercise capacity
- Brain structure, brain function, and neuroinflammation
- Circulating neurotrophic factors, markers of inflammation, and markers of cardiometabolic health
- Quadriceps strength and endurance
- Functional performance
- Autonomic cardiac function, resting heart rate and blood pressure
- Body composition
- Muscle structure and intramuscular adaptations of the m. vastus lateralis (only phase 1)
- Epigenetic modifications in muscle (only phase 1) and blood
- Impact of COPD, dyspnoea, and quality of life
- Exacerbations, hospitalizations, and mortality (phase 2)

We will also investigate the feasibility of the supramaximal HIIT and MICT interventions and the proportion of responders for each of the objectives to the interventions.

### Trial design

**Fig. 1:**
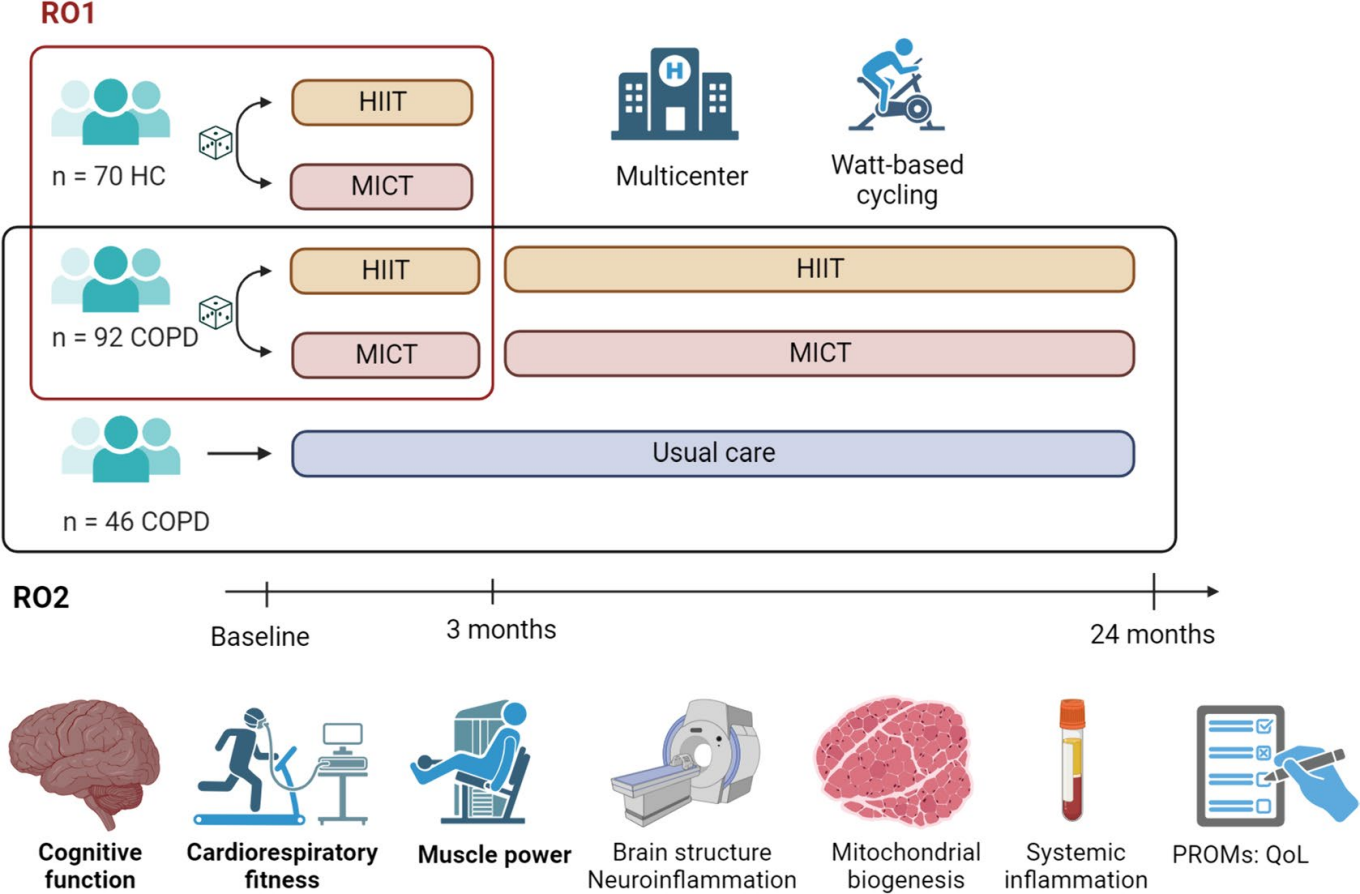
Visual representation of the COPD-HIIT trial. In phase 1 (RO1), supervised exercise training (supramaximal HIIT or MICT) will be performed two to three times per week for 12-weeks. In phase 2, participants with COPD will continue into a maintenance phase, exercising two times per week for 21-months. Legend: RO: research objective; HC: healthy controls; COPD: chronic obstructive pulmonary disease; HIIT, supramaximal high-intensity interval training, MICT, moderate intensity continuous training, PROMS, patient reported outcome measures, QoL, quality of life. Primary outcomes are indicated by bold text. Created with BioRender.com

**Fig. 2:**
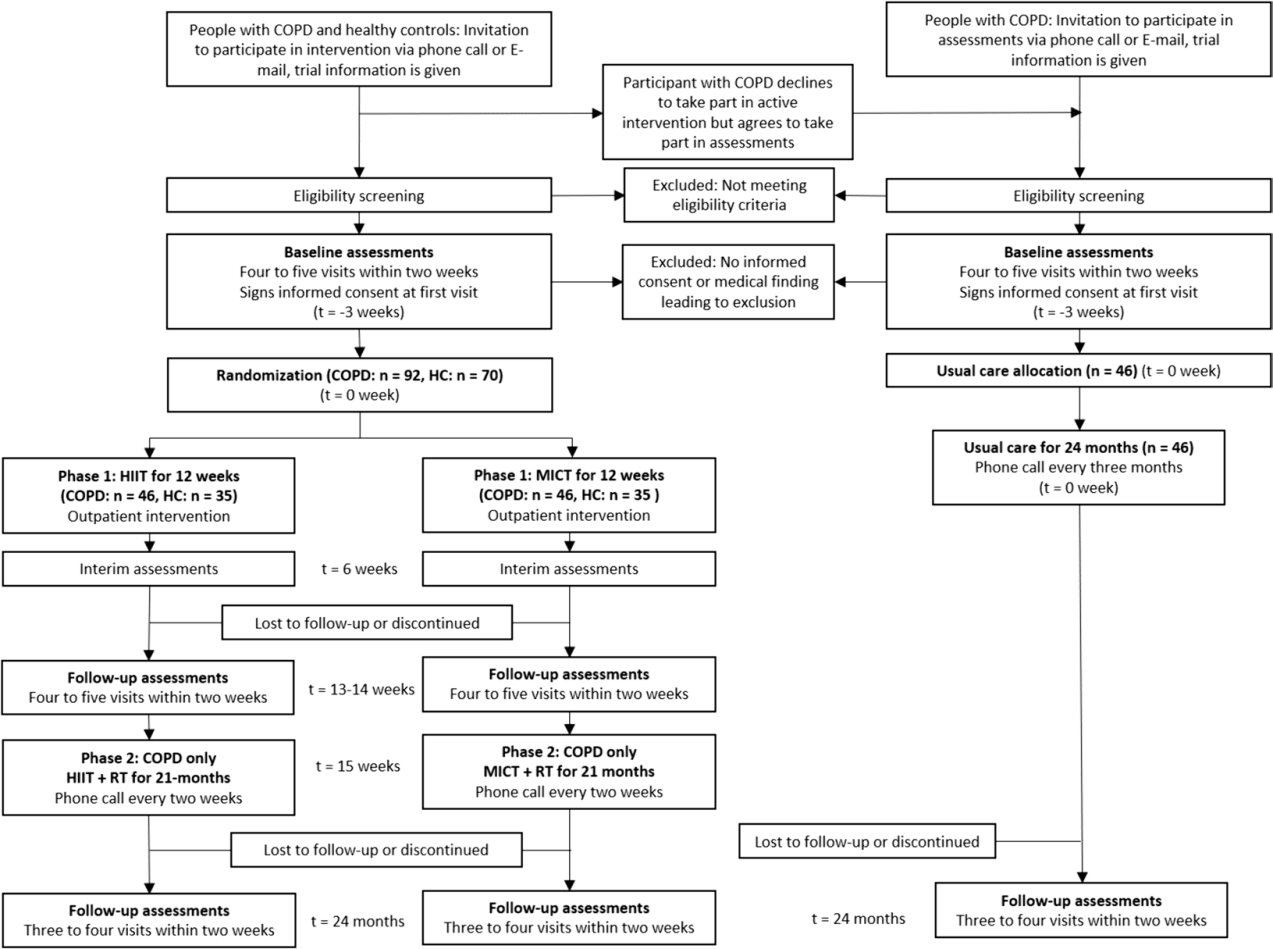
Overview of COPD-HIIT including participant flow. See Table 5 for specific information of each visit. COPD, chronic obstructive pulmonary disease, HC, healthy controls, HIIT, supramaximal high-intensity interval training, MICT, moderate intensity continuous training, RT, resistance training

COPD-HIIT is a prospective, multi-centre, randomized, controlled, parallel-group superiority trial with the assessor and statistician blinding, featuring a 1:1 allocation ratio and two separate phases. In phase 1, we will investigate the effects and mechanisms of a 12-week intervention with supramaximal HIIT compared to MICT in people with COPD and matched healthy controls. Upon completion of the initial 12-week intervention and follow-up assessments, people with COPD, but not healthy controls, will enter phase 2 of the trial. Phase 2 comprises a 21-month maintenance exercise program. Subsequently, exercise training (supramaximal HIIT or MICT) will be performed until a 24-month follow-up. A separate control group of people with COPD, receiving usual care only, will undergo assessments at baseline and 24 months. As the usual care arm is not included in the randomization, phase 2 is a partially randomized controlled trial. See Fig. 1 and Fig. 2 for an overview of the COPD-HIIT project design.

This protocol adheres to the Standard Protocol Items: Recommendations for Interventional Trials (SPIRIT) guidelines and its outcome extension [38–40] (see Table 5 for the SPIRIT Figure, and additional file 1 for the SPIRIT checklist). Intervention descriptions are further guided by the Template for Intervention Description and Replication (TIDieR) [41] (additional file 2) and the Consensus on Exercise Reporting Template (CERT) [42] (additional file 3). Publications from the COPD-HIIT project will follow the Consolidated Standard of Reporting Trials (CONSORT) statement [43] or relevant guidelines at the time of publication [44]. Publications including qualitative outcomes will also be guided by the Consolidated Criteria for Reporting Qualitative Research (COREQ) [45].

## Methods: settings and participants

### Study setting

Phases 1 and 2 of the COPD-HIIT trial will be performed at two centres, one in Sweden: Department of Community Medicine and Rehabilitation, Physiotherapy, Umeå University, Umeå, and Norrlands Universitetssjukhus (NUS, Umeå, Sweden) (site investigators AN (principal investigator), JJ and JV) and one in Belgium: Faculty of Rehabilitation Sciences, Hasselt University, Diepenbeek, and Ziekenhuis Oost-Limburg (ZOL, Genk, Belgium) and Jessa Ziekenhuis (Hasselt, Belgium) (site investigators CB (principal site investigator), JDB, DR, and DC). Enrolment began in November 2023, and we expect recruitment in phase 1 to be completed by the end of 2026. Thus, data collection of phase 2 is expected to be completed at the end of 2028. All trial centres will have qualified personnel including a medical doctor and health care professionals with experience in working with people with COPD. Principal site investigators have a Ph.D. and research experience in the research area. Trial centre facilities possess all the relevant equipment for performing relevant assessments and interventions (as described in the ‘ Interventions’ and ‘ Outcomes’ sections).

### Participants and recruitment

We will recruit participants with COPD via primary and specialist health care or advertisement. Matched healthy controls will be recruited via advertisement. All advertisements will have approval by the regional ethical board and will be disseminated via billboards, newspapers, local organization contacts, or targeted social media (e.g., Facebook). Each participant will receive financial compensation in an amount determined by the local centre and ethical board. If a participant would drop out, payments will be pro-rated based on the length of time stayed or the number of visits performed in the trial.

The planned distribution of participants per centre is as follows: in Umeå, approximately two-thirds (*n* = 138) of the participants will be recruited and in Hasselt the remaining third (*n* = 70) of participants. Subsequently, 92 individuals with COPD (HIIT: *n* = 31, MICT: *n* = 31, usual care: *n* = 30) and 46 HC (HIIT: *n* = 23, MICT: *n* = 23) are planned to be recruited in Umeå, while 46 individuals with COPD (HIIT: *n* = 15, MICT: *n* = 15, usual care: *n* = 16) and 24 HC (HIIT: *n* = 12, MICT: *n* = 12) are planned to be recruited in Hasselt. The enrolment period is expected to extend over approximately 24 months.

Written informed consent will be obtained by the assessors (AN, JJ, JDB, and DC) trained in Good Clinical Practice after all procedures have been explained, before the start of the trial during participants’ first test day.

### Eligibility criteria

The inclusion criteria are as follows:

- 60 years of age or older
- Independent in activities of daily living
- For people with COPD: post-bronchodilator spirometry confirmed COPD diagnosis (forced expiratory volume in one second (FEV_1_) to forced vital capacity (FVC) ratio < 0.70) [2].
- For people with COPD: symptomatic (COPD assessment test (CAT) ≥ 10 or modified Medical Research Council dyspnoea scale (mMRC) ≥ 2) or not being regularly physically active at a moderate or high intensity over the last year defined as not meeting WHO requirements for physical activity [46].
- For healthy controls: normal lung function

The exclusion criteria are as follows:

Movement-related, cardiovascular, neuromuscular, metabolic, skeletal and/or rheumatic conditions, and diseases that are unstable and/or prohibit exercise or tests, based on screening by a physician. For example, but not limited to:

- Musculoskeletal pain prohibiting participation in physical tests and exercise
- Recent myocardial infarction, coronary artery bypass grafting, angioplasty, or other cardiac events
- Uncontrolled arterial hypertension
- Pathological ECG-findings during CPET
- Other lung conditions, including but not limited to uncontrolled or very severe asthma, interstitial lung disease, lung cancer, pulmonary hypertension, pulmonary vascular disease, and pulmonary fibrosis.

Medical conditions and treatments with known effects on brain function and cognition, for example:

f) Previous trauma to the head with lasting cognitive or symptom-related issues
g) Physical or mental disabilities
h) Neurological condition (dementia, multiple sclerosis, stroke)
i) Psychiatric illness not including depression or general anxiety disorder
j) Severe cognitive impairment
k) Recent or current cancer diagnosis and treatment
l) For those accepting MRI or PET/CT: metal implants, pacemakers, claustrophobia, and other MRI-incompatible factors.
m) Inability to read or speak Swedish (Umeå participants), Dutch, French (Hasselt participants), or English (Umeå and Hasselt participants)

For people with COPD:

n) Comorbid conditions that limit exercise performance to a greater extent than the COPD diagnosis.
o) Currently participating in a pulmonary rehabilitation program or have been involved in pulmonary rehabilitation in the last 12 months.
p) Experienced a COPD exacerbation that led to a change in medication dosage or frequency in the preceding 6 weeks.

## Methods: interventions

### Phase 1

Eligible participants will be randomized to perform either supramaximal HIIT (intervention arm) or MICT (active control arm) on a stationary bicycle (Smart ZBike, Zycle, Valencia, Spain) coupled to a tablet. For both arms, training is performed two to three times per week for a total of 30 sessions (Table 1) using a group format with groups of four to eight participants at the same time. Group training will be conducted in designated rooms at a training facility. Visual supporting information (additional file 4, Figure S4-S5) will be displayed on the tablet and will include the session elapsed time, elapsed time per programmed phase, revolutions per minute (RPM), and target power output (TPO).

**Table 1:** Overview of exercise progression and progression criteria for supramaximal HIIT and MICT during phase 1

Block	Week	Session	HIIT % of MPO_6_	MICT % of MAP	General progression criteria		
	**0**	**Baseline testing**			**Criteria TPO escalation**	**HIIT**	**MICT**
**Mesocycle 1**: focus on intensity and duration (only MICT)	**1**	1	60%	50%	Able to maintain minimum RPM last interval/minute	80	60
		2	70%	55%	Exertion (RPE 6–20)	≤ 17	≤ 13
	**2**	3	80%	60%	Dyspnea (0–10)	≤ 7*	≤ 5
		4	#	¤	Note: Exertion and dyspnoea are assessed at the end of the last interval/minute		
	**3**	5	#	+ 2 min			
		6	#	¤			
	**4**	7	#	+ 2 min	**TPO progression criteria**		
		8	#	¤	**# Supramaximal HIIT % of workload**		
	**5**	9	#	+ 2 min	RPE ≤ 14 and dyspnoea ≤ 3	+ 5 to 10%	
		10	#	¤	RPE 15–17 and dyspnoea 4–7	+ 2.5 to 5%	
	**6**	11	#	+ 2 min	If RPE 18 or dyspnoea 8	Keep same	
		12	**CPET and BCST**		If RPE 19 or dyspnoea 9	-2.5 to -5%	
**Mesocycle 2**: increased frequency. Focus on increasing duration and intensity	**7**	13	#	¤	If RPE 20 or dyspnoea 10	-5 to -10%	
		14	#	¤	**¤ MICT % of workload**		
		15	#	¤	RPE ≤ 11 and dyspnoea ≤ 3	+ 5 to 10%	
	**8**	16	+ 1 interval	+ 2 min	RPE 12–13 and dyspnoea 4–5	+ 2.5 to 5%	
		17	#	¤	If RPE 14 or dyspnoea 6	Keep same	
		18	#	¤	RPE ≥ 15 or dyspnoea 7	-2.5 to -5%	
	**9**	19	+ 1 interval	+ 2 min	RPE ≥ 16 or dyspnoea ≥ 8	-5 to -10%	
		20	#	¤	Note: The highest rating informs the progression decision		
		21	#	¤			
	**10**	22	+ 1 interval	+ 2 min	**Duration progression criteria**		
		23	#	¤			
		24	#	¤	**Supramaximal HIIT duration**		
	**11**	25	+ 1 interval	+ 2 min	RPE ≤ 17 and dyspnoea ≤ 7	+ 1 interval	
		26	#	¤	If RPE 18 or dyspnoea 8	Keep same	
		27	#	¤	If RPE ≥ 19 or dyspnoea ≥ 9	- 1 interval	
	**12**	28	+ 1 interval	+ 2 min	**MICT duration**		
					RPE ≤ 13 and dyspnoea ≤ 5	+ 2 min	
		29	#	¤	If RPE 14 or dyspnoea 6	Keep same	
		30	#	¤	RPE ≥ 15 or dyspnoea ≥ 7	- 2 min	
	**13–14**	**Follow-up testing**			Note: The highest rating informs the progression decision. Duration is only increased if TPO is ≥ 80% MPO_6_ for HIIT, or 60% of MAP for MICT		

An overview of the intensity, duration, and their progression criteria to be used in phase 1. # indicates progression of supramaximal HIIT intensity based on criteria outlined in the table. ¤ indicates progression of MICT intensity based on criteria outlined in the table. HIIT intensities are expressed in % of MPO_6_ derived from the BCST while MICT intensities are expressed in % of MAP derived from a CPET. *BCST* Borg cycle strength test, *CPET* Cardiopulmonary exercise test, *HIIT* supramaximal high-intensity interval training, *MAP* maximal aerobic power, *MICT* moderate intensity continuous training, *MPO*_*6*_ maximal mean power output for six seconds, *RPE* rating of perceived exertion, *RPM* revolutions per minute, *TPO* target power output

^*^Based on our acute supramaximal HIIT preliminary data [17] and due to the short duration of the interval, a higher dyspnoea score cut-off is used compared to ERS guidelines [9]

Both protocols enable controlled and systematic adjustments of training intensity by means of standardized criteria to achieve a progressive overload (Table 1). The criteria in Table 1, including “general,” “TPO,” and “duration” should, as far as possible, be followed to ensure standardization across centres to facilitate replication and implementation in clinical settings. However, the instructor can override the suggested progression criteria if deemed necessary. For example, some individuals are so-called high raters and repeatedly rate high on [47] RPE and [47] CR-10 scales, which could result in a lack of progression or even de-escalation of TPO or duration. In those cases, the instructor can decide to override the criteria and decide to progress. In contrast, other individuals are “low raters,” which could result in a too rapid progression, and in those cases, the instructor can decide not to progress. Importantly, when the instructor does not follow the recommended progression criteria, the alteration will be noted and motivated. Other reasons for overriding the planned exercise training include day-to-day variations in well-being, e.g., due to extrinsic factors such as lack of sleep the night before a training session, muscle soreness, etcetera, could result in overriding the planned exercise session. The possibility to override planned exercise sessions and progression criteria should be used with caution.

Both exercise programs start with a 5-min warm-up and end with a 5-min cooldown, performed at an intensity corresponding to 30% of the maximal work rate achieved during a CPET (i.e., MAP, in watts (W)) with a self-selected pedalling cadence of 50–70 RPM. If a participant has a severe ventilatory limitation or very low exercise capacity, defined as a CPET duration < 6 min, a 2-min warm-up will be utilized. The 12-week exercise intervention will be divided into two 6-week mesocycles (Table 1) and the training load (intensity and/or duration) regulated to optimize exercise training prescription and health benefits.

### Phase 1: interim assessments

During week 6, one training session will be replaced by interim assessments. Participants will perform a CPET and, after at least 1 h of rest, a Borg cycle strength test (BCST) [15, 48]. These assessments will provide updated MAP and maximum mean power output for 6 s (MPO_6_) data to optimize exercise intensities for MICT and HIIT in the following mesocycle. If a participant is already exercising at or above the intensity estimated from the interim assessment, the higher training intensity load will be kept. We refer to the outcomes section for further information of these assessments.

### Phase 1: supervision

All exercise sessions will be held and supervised by an experienced health care professional, such as physiotherapists, exercise physiologists, or other health care professional with equivalent expertise. All intervention providers will receive training on exercise intervention protocols to ensure standardization among centres. Instructors will give preparatory information during the training sessions, e.g., “In five seconds, increase your pedalling cadence up to 85 RPM”. Encouragement will be given during the sessions but be kept modest to not push the participants to exceed the intensity.

### Intervention arm: supramaximal HIIT

Following the warm-up, participants will perform 10 min of supramaximal HIIT. Supramaximal HIIT is performed as 10 × 6 s intervals against an individualized TPO. Since it takes approximately 6 s for the bike to apply the necessary breaking resistance to reach the TPO, each interval is programmed to last 12 s with a 48-s recovery between intervals. The first 20 s of the recovery is passive, while the latter 28 s are active at 30% of MAP. Target pedalling cadence for the intervals is 80–90 RPM and 50–70 RPM for the active recovery. Thus, regardless of individual capability, pedalling cadence will be even and controlled throughout the training sessions. If a participant is not capable of pedalling at 80–90 RPM, a lower cadence of 60–70 RPM will be allowed. Would a participant not be able to complete a 12-s interval, they will be instructed to not pedal during the next interval but only during the 48-s recovery phase. Then, the participant can continue with the following intervals or every-other interval, as based on instructor and participant judgement.

Including the warm-up and cool-down, the session duration is 20 min. Initial TPO for the supramaximal HIIT intervals is set at 60% of the MPO_6_ derived from a BCST (see “ Outcomes” section). The intensity and number of intervals (total training session duration) will be increased according to an escalation procedure (Table 1). The initial TPO of 60% of MPO_6_ is considered a familiarization session, and the intention is to increase the intensity to 80% of MPO_6_ in session 3 if the general progression criteria outlined in Table 1 are fulfilled.

### Active control arm: MICT

Participants in the active control arm will perform MICT designed following the current guidelines for moderate-intensity continuous exercise in both people with COPD and healthy older adults [9]. The MICT includes 20 min at a TPO of 50% of MAP with a target pedalling cadence of 60–70 RPM. Would a participant not be able to keep the target cadence, the intensity will be reduced to approximately 50% of the TPO until the cool-down phase.

Including warm-up and cool-down, the total session duration is 30 min. The intensity and duration of the 20-min exercise phase will be progressed according to general methods for MICT exercises. The initial TPO of 50% of MAP is considered a familiarization session, and the intention is to increase the intensity to 60% of MAP in session 3. As for supramaximal HIIT, the escalation of TPO in the MICT will be based on the criteria outlined in Table 1. The MICT has been chosen as an active comparator because we want to compare supramaximal HIIT to the current guidelines [9] for endurance exercise in both people with COPD and healthy older adults.

### Phase 2

#### Maintenance program

Following the first 12-week training period, participants with COPD will enter a 21-month maintenance phase (Table 2). After 2 weeks with outcome assessments and 1 to 2 weeks of rest, they will continue to exercise using the same training modality (supramaximal HIIT or MICT) as during the first 12 weeks but can select between three different settings to continue their training: ‘ Home’, ‘ Outpatient’, or ‘ Mix’ setting. The participant can, at any time during the maintenance period, change between the ‘ Home’, ‘ Outpatient,’ or ‘ Mix’ setting when conducting their endurance training. A description of each setting is provided below. Importantly, these different settings should not be seen as different trial arms. That is, irrespective of whether a participant in the supramaximal HIIT or MICT group selects to continue their training at home, in an outpatient setting or in a mixed setting, data analysis will be conducted per allocated trial arm (supramaximal HIIT, MICT, usual care).

**Table 2:** Overview of exercise progression and progression criteria for supramaximal HIIT and MICT during phase 2

Block	Week	Session	HIIT	MICT	HIIT-Walk	MICT-Walk	General progression criteria		
	**13–14**	**Testing after 12-week intervention**					Criteria for progression are evaluated every two-week period by the research group		
**Mesocycle 1:** Focus on duration and intensity	15–18	1–8	#	**¤**	##	¤¤			
	19–22	9–16	+ 1–2 intervals	+ 2–4 min	+ 1–2 intervals	+ 2–4 min	**HIIT (outpatient, home, or mix)**		
	23–26	17–24	#	**¤**	##	¤¤	TPO (#) / duration		
	27–30	25–32	+ 1–2 intervals	+ 2–4 min	+ 1–2 intervals	+ 2–4 min	RPE ≤ 14 and dyspnoea ≤ 3 RPE 15–17 and dyspnoea 4–7 RPE 18 or dyspnoea 8 RPE 19 or dyspnoea 9 RPE 20 or dyspnoea 10	+ 5 to 10% or 2 intervals + 2.5 to 5% or 1 interval Keep same -2.5 to -5% or 1 interval -5 to -10% or 2 intervals	
	31–34	33–40	#	**¤**	##	¤¤			
	35–38	41–48	+ 1–2 intervals	+ 2–4 min	+ 1–2 intervals	+ 2–4 min	Note: Increase of HIIT intervals: max + 10 intervals for a total of 20 intervals. If reached, only intensity will increase		
	39–42	49–56	#	**¤**	##	¤¤	**MICT (outpatient, home, or mix)**		
	43–46	57–64	+ 1–2 intervals	+ 2–4 min	+ 1–2 intervals	+ 2–4 min	TPO (**¤**) / duration		
	47–50	65–72	#	**¤**	##	¤¤	RPE ≤ 11 and dyspnoea ≤ 3 RPE 12–13 and dyspnoea 4–5 RPE 14 or dyspnoea 6 RPE 15 or dyspnoea 7 RPE ≥ 16 or dyspnoea ≥ 8		+ 5 to 10% or 4-min + 2.5 to 5% or 2-min Keep same -2.5 to -5% or 2-min -5 to -10% or 4-min
	51–54	73–80	+ 1–2 intervals	+ 2–4 min	+ 1–2 intervals	+ 2–4 min			
**Mesocycle 2:** Focus on intensity	55–58	81–88	#	**¤**	##	¤¤	Note: Increase of MICT duration: max + 30 min, for a total of 50 min. If reached, only intensity will increase		
	59–62	89–96	#	**¤**	##	¤¤	**HIIT-Walk (home or mix)**		
	63–66	97–104	#	**¤**	##	¤¤	Intensity (##) / duration		
	67–70	105–112	#	**¤**	##	¤¤	RPE ≤ 14 and dyspnoea ≤ 3 RPE 15–17 and dyspnoea 4–7 RPE 18 or dyspnoea 8 RPE ≥ 19 or dyspnoea ≥ 9	↑speed or + 2 intervals + 1 interval Keep same ↓ speed or -1 interval	
	71–74	113–120	#	**¤**	##	¤¤			
	75–78	121–128	#	**¤**	##	¤¤	Note: Increase of HIIT-Walk intervals: max + 10 intervals, for a total of 20 intervals. If reached, only intensity will increase		
	79–82	129–136	#	**¤**	##	¤¤	¤¤ **MICT-Walk** **(home or mix)**		
	83–86	137–144	#	**¤**	##	¤¤	Intensity (¤¤) / duration		
	87–90	145–152	#	**¤**	##	¤¤	RPE ≥ 11 and dyspnoea ≤ 3 RPE 12–13 and dyspnoea 4–5 RPE 14 or dyspnoea 6 RPE ≥ 15 or dyspnoea ≥ 7	↑speed or + 4 min + 2 min Keep same ↓ speed or -2 min	
	91–94	153–160	#	**¤**	##	¤¤			
	95–98	161–168	#	**¤**	##	¤¤	Note: Increase of MICT-Walk duration: max + 30 min, for a total of 50 min. If reached, only intensity will increase		
	99–102	169–176	#	**¤**	##	¤¤			
	**103–104**	**Testing after 21-month maintenance**							

An overview of the intensity, duration, and their progression criteria to be used in phase 1. The actual number of weeks displayed in the table do not include the one to two rest weeks after phase 1. # and ## indicates progression of intensity in supramaximal HIIT and HIIT-Walk, respectively, based on criteria outlined in the table. ¤ and  ¤ ¤ indicates progression of intensity in MICT and MICT-Walk, respectively, based on criteria outlined in the table. *HIIT* supramaximal high-intensity interval training, *MICT* moderate-intensity continuous training, *TPO* target power output

#### Home

Participants with COPD that select to continue with endurance training in a home setting will be provided with a cycle ergometer (Smart ZBike, Zycle, Valencia, Spain) for unsupervised supramaximal HIIT or MICT. Alternatively, if a participant would like to continue at home but does not want to receive a cycle ergometer, they can continue with walking-based HIIT or MICT (HIIT-Walk or MICT-Walk). The HIIT-Walk and MICT-Walk build on the same principles as the supramaximal HIIT and MICT exercise performed on a cycle ergometer but are modified to be suitable during walking. See Tables 2 and 3 for details.

**Table 3:** Description of Endurance Training (ET) in line with TIDieR and CERT guidelines

Variable	Supramaximal HIIT	MICT	HIIT-Walk	MICT-Walk
1. ET objective	The ET objective for all interventions is to improve peak VO_2_ on a CPET and exercise duration on a CWRT.			
2. Training device / type of exercise	Cycling on a stationary cycle ergometer (Smart Zbike, Zycle, Valencia, Spain)		Outdoor walking	
3. Supervision and delivery	First 12 weeks and outpatient last 21 months: Group of 4-5 individuals under the supervision of a trained instructor^a^, face-to-face delivery / Home or mix last 21 months: unsupervised cycling, individual, phone call delivery every two weeks		Unsupervised walking training, individual, phone call delivery every two weeks	
4. Setting	First 12 weeks: outpatient (local gym or rehab center) / Last 21 months: Home, outpatient (local gym or rehab center) or mix (local gym or rehab center + home)		Home	
5. Adjunct	First 12 weeks: none / Last 21 months: RT (see Table 4)		RT (see Table 4)	
6. Program duration	12 weeks + ≈ 21 months		≈ 21 months	
7. Frequency of sessions	Week 1-6: 2x/week / Week 7-12: 3x/week / Week 15 to 102: 2x/week		Week 15 to 102: 2x/week	
8. Exercise selection	10×6 sec intervals against an individualized intensity with 48 sec recovery^b^ between intervals (20s passive, 28s active) for 10 min	Continuous cycling against an individualized intensity for 20 min	10×10 sec intervals at an individualized maximal walking speed with 50 sec of active rest between intervals for 10 min	Continuous walking at an individualized intensity for 20 min
9. Warm-up / cool-down	5 min at 30% of MAP at 50-70 RPM^c^		5 min at a walking speed at Borg dyspnea <3	
10. Intensity	Start intensity of 60% of MPO_6_ with aim of increasing up to 80% of MPO_6_ in session 3.	Start intensity at 50% of MAP with aim of increasing up to 60% of MAP in session 3.	Start intensity at a walking speed as tolerated at Borg dyspnea 4-8 at the end of the last interval	Start intensity at a walking speed as tolerated at Borg dyspnea 4-6 at the end of the last minute
11. Exercise volume	Exercise volume will be evaluated by: duration (min:ss), training load in kilojoule (intensity × duration), duration at intensity above MAP (mm:ss)		Cannot be accurately calculated	
12. Velocity of muscle action	80-90 RPM	60-70 RPM	Self-determined walking speed at Borg dyspnea 4-8 and 4-6 for HIIT-Walk and MICT-Walk, resp.	
13. Rest periods between series / type of rest incl. intensity	20 sec passive, 28 sec active rest at 30% of MAP between intervals	N/A	50 sec of active rest at Borg dyspnea < 3	N/A
14. Rest / sequence between exercises	If ET and RT are performed on the same day, a minimum of 15 min rest between ET and RT, and ET is performed first			
15. Recovery	At least 48h rest between sessions			
16. Attendance (% of sessions) / Adherence^d^	Completion rate, attendance rate, and adherence to exercise duration and intensity will be obtained.			
17. Specificity	Variables 6-16 are designed to achieve the ET objective (Variable 1)			
18. Progressive overload	Progression/overload criteria are seen in Table 1- 2		Progression/overload criteria are seen in Table 2	
19. Variation	Non-linear block periodization			
20. Individuality	Designed to lower the ventilatory demand, based on BCST data to set intensity	Designed per ATS/ERS guidelines^9^, based on CPET data to set intensity	N/A	N/A

*CPET *cardiopulmonary exercise test,* CWRT *constant-workload test,* ET* endurance training, *ATS/ERS* American Thoracic Society/European Respiratory Society, *MICT* moderate intensity continuous training, *HIIT* high-intensity interval training, *MPO*_*6*_ maximal mean power output for six seconds, *N/A* Not applicable, *RPE* rating of perceived exertion, *RPM* revolutions per minute, *RT* resistance training

^a^The instructor education consists of written, oral and visual instructions on how to perform HIIT & MICT exercises, and instructors are qualified health professionals (e.g. physiotherapists, exercise physiologists)

^b^Since it takes approximately six seconds for the bike to apply the necessary breaking resistance to reach target intensity, each interval is programmed to last 12 seconds with a 48 second recovery between intervals

^c^For those that cannot complete a CPET test according to guidelines, the warm-up will initially consist of 3-min seated rest and 2-min active warm-up as described for variable 9

^d^For those performing HIIT, MICT, HIIT-Walk or MICT-Walk during phase 2, attendance/adherence will be collected from a self-reported diary and followed up in the standardized phone calls every 14 days

#### Outpatient

Participants with COPD that select to continue in an outpatient setting will perform HIIT and MICT training in the same manner as during the initial 12-week period, supervised in small groups of 4–8 participants (see Tables 2 and 3).

#### Mix

For participants with COPD that select to continue in a mixed setting, home-based sessions will be combined with one supervised outpatient exercise session every 2 weeks (replacing one of the home-based sessions during these weeks). For example, for 1 month, the participant will complete six unsupervised home-based sessions and two supervised outpatient sessions (see Tables 2 and 3).

#### Resistance training (RT)

Irrespective of the originally assigned group (supramaximal HIIT or MICT) and chosen setting, all participants will also perform a RT regime. The RT regime will consist of ten lower- and upper-body exercises designed following American College of Sports Medicine guidelines [49] and other relevant RT literature for increasing muscular strength, endurance, and power. The goal of the RT regime (strength, endurance, or power) will change monthly. See Table 4 and additional file 5, Figure S1-S3 for details of the RT regime.

**Table 4:** Description of resistance training (RT) in line with TIDieR and CERT guidelines

Variable	Description
1. RT objective	The aims of the RT exercises are to improve limb muscle strength, power, and endurance
2. Training device and type of exercise	All exercises will be performed using the body weight of the participant and/or with elastic bands (Thera-Bands®, The Hygienic Corporation, Akron, OH, USA). The participants will be provided with the elastic bands. If performed in an outpatient setting, available RT equipment will be used. (Umeå: leg press, leg extension, chest press and lat pull-down machines, Nautilus, Core Health and Fitness LLC, Vancouver, WA, USA. Hasselt: lat pull-down and leg press machines, Enraf–Nonius B.V. Echt, The Netherlands). At both centres, Free-weight dumbbells, (Ziva, Raleigh, NC 27606, USA) will be used for elbow and shoulder flexion)
3. Provider & Location	Each session will be performed unsupervised in the home setting of the participant or in an outpatient setting (local gym or rehab centre) or in an outpatient setting (local gym or rehab centre). The RT is performed individually and delivered via a two-weekly phone call
4. Warm-up	No specific warm-up will be conducted before start of the RT
5. Muscle actions	Exercises will be performed using dynamic concentric/eccentric muscle actions
6. Exercise selection & order	An exercise battery of ten exercises will be available. The participants will be instructed to choose and perform four exercises in each exercise session within a two-week period. A sit-to-stand exercise should always be included (if performed in an outpatient setting a leg press or knee extension should always be included), but the participant should select three additional exercises each session. A pre-determined exercise order will be provided in the following order: sit to stand (m. quadriceps), calf raises (m. triceps surae), latissimus row (m. latissimus dorsi), chest press (m. pectoralis major, m. deltoid anterior), elbow flexion (m. biceps brachii), and shoulder flexion (m. deltoid anterior). If requested by the participant, additional exercises can be added or removed from the 10 exercises that are initially provided. However, the sit-to-stand exercise should always be included. See additional file 5 Figure S1-S3 for more information, including illustrations, start and end positions, and instructions for exercise executions
7. Intensity (loading)	For elastic band/equipment exercises, to achieve adequate loadings, the elastic band will be stretched/or weight added so that the participant can do between 8–12 repetitions (8–12 RM) for muscular strength and between 15–25 repetitions (15–25 RM) for muscular endurance. For muscle power, [50, 51] the same load as for muscular endurance will be used, but the participant will perform 8–12 repetitions. Thus, although performing 8–12 repetitions for power, it should not be at 8–12 RM load, the patient should be able to perform additional repetitions with ease and the focus is on speed of contraction (see variable 9). For body-weight exercises (sit to stand, calf raises), three different levels will be available (level 1, 2, 3). The start-level will be individualized based on an individuals’ muscle function and RT experience. If needed additional levels will be provided
8. Exercise volume	Two sets per exercise will be performed. We will not be able to calculate exercise volume accurately for home exercises. For outpatient exercises, exercise volume will be calculated as repetitions × load
9. Velocity of muscle action	All muscle strength and endurance exercises will be instructed to be performed using a moderate velocity (1:1 s in the concentric and eccentric phase respectively), for two sets per exercise. For muscle power, the exercises should be performed at a maximal individual speed for the concentric phase of the movement – and 2–3 s in the eccentric phase
10. Rest periods between sets	For muscle strength and endurance, we will use 1–2 min rest between sets, while a minimum of 3 min of rest between sets is used for muscle power
11. Rest periods / exercises	Rest periods between exercises will be 4 min
12. Recovery	At least 48 h rest between sessions
13. Cool down/ stretching	No specific cool-down will be applied
14. Frequency and program duration	Two times per week for ≈ 21 months
15. Adherence	Self-reported attendance and adherence to predefined number of exercises and sets will be obtained
16. Specificity	Variables 4–9 are designed in line with ACSM guidelines and previous research to achieve the RT objectives
17. Progressive overload	Progressive overload will be achieved by an individual progression of each exercise, i.e., for muscle strength (target 8-12 RM). If a participant could not do 8 repetitions, the resistance is too great, and the resistance is reduced. If the participant could do more than 12 repetitions, the exercise is too easy, and the participant should increase the resistance/weight or the level of the exercise. To increase the loading (weight) on the elastic band exercises, the participant is instructed to "shorten" the length of the elastic band by moving his/her grip closer to the origin of the elastic band. E.g., in the shoulder exercise by moving your grip further down on the elastic closer to your feet. To decrease the loading the participant should "lengthen" the elastic band by moving his/her grip further from the origin of the elastic band. To increase/decrease the difficulty of the sit to stand or the calf raise exercises you change the level (Level 1–2-3). If using equipment, the loading is increased with 2–10%. For muscular endurance, the same principles would be applied but the repetition zone would be 15-25RM instead of 8-12RM; For muscle power, the progression of loading used will follow the muscular endurance progression. Participants will be instructed to change between muscular strength, endurance, and power every month. Every two weeks, progression will be guided based on 1) if target repetition range is reached or not, and 2) perceived heaviness ratings [47]CR-10) of the 2nd set of every exercise from the last performed session in the previous two weeks
18. Periodization	No pre-planned periodization of program variables will be included
19. Tailoring	Tailoring of the resistance exercises will be dependent on whether the goal is to increase muscular strength, endurance, or power. See e.g., variable 7 for how the loading is altered depending on the goal

*ACSM* American College of Sports Medicine, *RM* repetition maximum, *RT* resistance training

#### Standardized phone call and letters via post

Participants with COPD will also, irrespective of the originally assigned group (HIIT or MICT) and chosen setting, receive a phone call once every 14 days. Using a standardized call protocol (see additional file 4), we will gather the necessary information to progress training intensity and information about attendance and adherence to the exercise intervention. Every third month, information about symptoms of exacerbations will also be collected via phone call (see additional file 4). Participants with COPD will also be provided with a diary where they can document their training sessions and write down additional information, e.g., change in medication. Additionally, questions on health status (CAT) and disease-specific quality of life (chronic respiratory disease questionnaire (CRQ)) will be collected every third month by sending a letter with the questionnaires via the post that will be self-administered or by a phone call.

#### Passive control group: usual care

The passive control group will receive usual care alone and a standardized phone call (see additional file 4) every 3 months including assessment of symptoms of exacerbations. Health status (CAT) and disease-specific quality of life (CRQ) will be assessed by sending a letter with the questionnaires via the post for self-administration or by a phone call. We will match the participants in the usual care group to those randomized to HIIT or MICT by age, sex, disease severity (GOLD A/B/E), educational level, and physical activity.

### Criteria for discontinuing the intervention or modifying allocated interventions

Criteria for discontinuing the intervention for a given trial participant are adverse events, severe injuries, or illness preventing to participate in the intervention or on participant request. In the case of discontinuation, trial participants will be retained in the trial whenever possible to enable follow-up data collection which can be extended up to a maximal of 2 weeks. Modifications of the allocated interventions are described below in the adherence section.

### Interventions—adherence

#### Phase 1

To promote adherence to the interventions during phase 1, efforts will be made to design the training sessions to be pleasant for participants. We strive for easy access to training facilities and adequately sized training groups in a comfortable setting using appropriate equipment with personal monitoring from experienced health care professionals, such as physiotherapists, exercise physiologists, or other health care professionals with equivalent expertise. The motivational principle of ‘visual cues and feedback’ will be used as the participant receives continuous visual supporting (additional file 4, Figure S4-S5) information during exercise on the tablet through the Kinomap exercise training application (Kinomap, Douai, Nord-Pas-de-Calais, France), and the motivational principle of ‘verbal cues and feedback’ given by the instructor will be used to facilitate the exercise sessions (as described under ‘Supervision’). The importance of adherence to the interventions will be informed before and during the trial. Trial participation will, if possibly, be planned to avoid breaks due to holidays and festive seasons.

Attendance to the intervention will be documented by the instructor conducting the training sessions at each centre. If a participant misses a training session without notice, an instructor will contact the participant by phone the same day. If needed due to missed sessions, the intervention period can be extended by up to 1 week, or if needed to reach a 75% attendance rate (22 sessions) up to 2 weeks, given that the participant agrees to the extension. Adherence to the prescribed exercise training (duration, intensity, RPM) will be documented by the instructor and via the Kinomap exercise training application.

#### Phase 2

To promote adherence to interventions during phase 2, the motivational principle of ‘preferred environments’ is used where participants with COPD will be able to select in which setting they wish to continue their exercise training, i.e., home-based, outpatient, or mixed setting. Furthermore, they can at any time during the 21-month maintenance period change between the different settings.

The individuals with COPD continuing in a home-based or mixed setting will receive a home visit at the start of phase 2 to deliver the necessary equipment and to receive instructions on how to perform the endurance and resistance training exercises at home. Since the endurance exercise is similar to phase 1—the primary focus of the instructions is on the resistance exercises, which are instructed orally and/or practically. Practical demonstration of at least one upper and one lower extremity exercise will always be performed. All participants will receive a training diary that is specific to their group allocation (supramaximal HIIT or MICT), [47] RPE 6–20 and [47] CR-10 rating scales, and elastic training bands. If a participant uses a bike at home, they will also be provided with a dedicated booklet on how to handle the bike and guidance on utilizing the Kinomap application. During office hours, the instructor will also be reachable to provide support over the phone if needed.

Additionally, the motivational principle of ‘visual cues and feedback’ (additional file 4, Figure S4-S5) will be used as the participant receive continuous visual supporting information during exercise through the Kinomap application (as described under ‘Interventions’), while the motivational principle of ‘verbal cues and feedback’ given by the instructor to facilitate the exercise sessions will only be applicable when the participant chooses the outpatient setting (as described under ‘Supervision’). The motivational principle of ‘visual cues’ will also be used for the resistance training via the use of images of all the exercises (as described in additional file 5. – Figure S1-S3). The participants will also receive a standardized follow-up call at the start of every 2-week period, including pre-determined questions on adherence to the exercise interventions (duration, intensity, RPM). The instructor will also be able to access the performed exercise training via the Kinomap exercise training application. The feedback given by the instructor can also be considered to be a motivational strategy using the principle of ‘verbal feedback’. The intervention period in phase 2 will not be extended to account for missed sessions in any of the settings.

### Relevant concomitant care permitted or prohibited during the trial

#### Phase 1

Participants are encouraged to continue with their regular everyday physical activity but are prohibited to participate in other organized training programs during the 12-week trial period.

#### Phase 2

Participants are encouraged to continue with their everyday physical activity and are allowed to enrol in a pulmonary rehabilitation (PR) program if that would be offered during the 24-month trial period. Participants are encouraged to discuss the uptake of a PR program with the trial team. For participants in the HIIT or MICT arm, the trial team will make sure the participant can still proceed with their allocated endurance (supramaximal HIIT or MICT) modality within the PR program (if the PR program is attended within NUS or ZOL). For participants in the passive control group, enrolment in a PR program cannot be denied and will not result in exclusion from the trial, but these participants will not be included in the per-protocol analysis.

## Methods: data collection, outcomes, and analysis

### Standardisation

Assessors will have previous experience or be trained in the included outcome assessments before commencing the trial. Protocols will be developed to standardize procedures. All tests will be performed using equipment that is available at each trial centre, which will be standardized to the greatest extent possible via standard operating procedures. Efforts will be made to ensure within-participant as well as between-participant outcome assessments are performed at the same time of day to minimize the effects of diurnal variations.

### Screening procedure

Eligibility screening will start with a telephone call (Umeå and Hasselt) or an in-person conversation (Hasselt) by a member of the research team to each participant to assess the inclusion and exclusion criteria. The responsible pulmonologist at each trial centre will, after consent, screen the medical record of the participant to assess eligibility. If the participant is eligible based on the screening, the participant will receive an accelerometer (DynaPort®, McRoberts BV, The Netherlands) to document his/her physical activity level. The participant will be instructed to wear the accelerometer on the lower back during seven consecutive days. The quantity of physical activity will be assessed using the mean number of steps per day and time spent in at least moderate-intensity physical activities. Accelerometer data consisting of ≥ 8 h/day wear time with ≥ 4 days of measurements will be considered valid [52]. In people with COPD, self-reported physical activity level measured by indicator questions from the National Board of Health and Welfare in Sweden will be used to determine eligibility when people with COPD are non-symptomatic based on mMRC dyspnoea scale or CAT. In controls, the physical activity data will be used to assess matching potential together with age and sex, and thus eligibility, and for baseline physical activity level determination. Next, the participants proceed to two–three weeks of baseline assessments where abnormal findings on examinations can lead to non-eligibility and thus exclusion.

### Assessments only at baseline

Assessments only at baseline are measures with the purpose to characterize the participants and are only considered outcomes for cross-sectional comparisons between groups at baseline.

- Pulmonary function testing will be performed including spirometry, lung volumes, and diffusion capacity for carbon monoxide according to ATS/ERS guidelines [53–55]. At follow-up testing, a spirometry is performed for up-to-date pulmonary data during cycling tests.
- Anthropometrics: to measure height, participants will stand against a stadiometer without shoes. Body weight loss and malnutrition will be assessed using the Self-MNA® Mini Nutritional Assessment. The Self-MNA® consists of 5 questions related to food intake, weight loss, mobility, recent psychological stress or acute disease, neuropsychological features (dementia/sadness), and three anthropometric assessments (height, weight, and calf circumference), resulting in a calculated score (maximal 14 points). Based on the score, the participant is categorized as malnourished (0–7 points), at risk of malnutrition (8–11 points), and normal nutritional status (12–14 points). The Self-MNA® is the self-report version of the MNA short form that has been previously used in COPD [56], validated against the full MNA [57], and is advised to use as a nutritional screening tool by the European Society for Clinical Nutrition and Metabolism guidelines [58]. The administration of the Self-MNA® takes less than 5 min to complete.
- Information on smoking status and habits, pack years, exacerbations, and hospitalisations during the last 12 months, medical history, comorbidities, pharmacological treatments (any changes in medication will also be collected during the trial period), educational level, and history of risk factors for COPD (i.e., biomass exposure, occupational exposure, and host factors).
- Information on training status: any previous training experience, aerobic training experience, and specifically, exercise habits (type, frequency, intensity) in the last 12 months.
- The modified medical research council (mMRC) dyspnoea scale (0–4, arbitrary units) will be used to assess baseline dyspnoea [59]. The mMRC dyspnoea scale is used to evaluate the degree of baseline functional disability in patients with respiratory diseases [59]. The scale consists of five levels ranging from 0 to 4, each representing a different level of dyspnoea where grade 0 is the lowest degree of functional disability, and 4 the highest. The scale is easy and efficient to use and widely used in the COPD literature and is integrated with the Global Initiative for Chronic Obstructive Lung Disease (GOLD) clinical classification scheme [2]. The mMRC takes 2–3 min to complete.
- Physical activity (see description above).

### Outcomes

Primary and secondary outcomes are assessed at baseline, 12 weeks and 24 months if not stated otherwise. The analysis metric is change from baseline at 12 weeks (phase 1) and change from baseline at 24 months (phase 2), if not stated otherwise. Outcomes will be aggregated as mean ± SD or median (interquartile range) depending on distribution of data, unless otherwise specified.

Before, during and after all physical tests, the level of dyspnoea and leg fatigue (0–10, arbitrary units) on the [47] CR-10 scale [47] and perceived exertion (6–20, arbitrary units) on the [47] RPE scale [60] will be assessed. An overview of outcome assessments is outlined in Table 5 (SPIRIT Figure).

**Table 5:** Time schedule of enrolment, assessments and visits for participants

		Screening	Baseline					Allocation	Phase 1	Interim	Follow-up assessments				Phase 2	Follow-up assessments			
Timepoint		-T6	-T5	-T4	-T3	-T2	-T1	0		T1	T2	T3	T4	T5		T6	T7	T8	T9
Timeline (weeks)			-3 to -1					0	0-12	6	13-14				15-102	103-104			
**ENROLMENT**:																			
Eligibility screening		X																	
Informed consent			X																
Allocation^c^								X											
**INTERVENTIONS**:																			
HIIT/MICT/Usual care									X						X				
**ASSESSMENTS**:																			
**Visit 1 - Baseline only**																			
Physical activity		X																	
mMRC and self-MNA		X^a^	X																
Lung function			X																
Medical history and demographic data			X																
**Visit 1**																			
CPET and BCST			X							X	X					X			
MoCA, CAT and CRQ^d^		X^b^	X								X^f^				X	X			
Quadriceps function tests			X								X					X			
**Visit 2**																			
Body composition				X								X					X		
Cardiovascular function				X								X					X		
Global cognitive function				X								X					X		
Constant work-rate test				X								X					X		
HADS and EQ-5D-5L				X								X					X		
5-time sit-to-stand and stair climb power test				X								X					X		
**Visit 3**																			
Blood sampling					X								X					X	
Blood pressure					X								X					X	
Muscle biopsy^c^					X								X						
**Visit 4**																			
Brain magnetic resonance imaging						X								X				X	
**Visit 5**																			
PET/CT^e^							X												X
I**nterventional**																			
Feasibility^c^									X						X				
Adverse events									X						X				
Exacerbations / hospitalisations / mortality									X						X				

Schedule of enrolment, interventions and assessments. The number of weeks can vary slightly depending on schedueling and thus time needed for follow-up assessments (2-3 weeks) and weeks of rest before starting the phase 2 (1-2 weeks). The schedule is representative for participants in Umeå, Sweden, minor differences exist for participants in Hasselt, Belgium, due to logistics

*HIIT* supramaximal high-intensity interval training, *MICT* moderate intensity continuous training, *mMRC* Medical Research Council Dyspnea Scale, *MoCA* the Montreal Cognitive Assessment test, *CPET* cardiopulmonary exercise test, *BCST* Borg cycle strength test, *CAT *COPD assessment test, *CRQ* Chronic Respiratory Disease Questionnaire, *HADS* Hospital Anxiety and Depression Scale, *MNA* Mini-Nutritional Assessment, *PET/CT* positron emission tomography/computed tomography

^a^only mMRC is used for screening

^b^only CAT is used for screening

^c^not for usual care group

^d^not for HC group

^e^only for those enrolled at Umeå, Sweden

^f^(superscripted) MoCA is only performed at baseline at 24-months

### Primary outcomes

Phase 1 and phase 2 include the same three primary outcomes. These are multiple primary outcomes and not co-primary outcomes. Each outcome is deemed sufficient on its own to establish intervention efficacy. Multiple primary outcomes are selected in the COPD-HIIT project as we intend to determine the effects on multiple extrapulmonary manifestations of the disease which cannot be adequately quantified using a single primary outcome [61–63].

#### Global cognitive function

Global cognitive function will be assessed as the z-score determined by the performance on five tests from the Cambridge Neuropsychological Test Automated Battery (CANTAB) and the trail making test [64] (TMT). To create a global cognitive score, we will convert the six separate test scores from the CANTAB tests and the TMT to a composite z-score (Table 6).

**Table 6:** Overview of cognitive test battery structured per cognitive domain

Cognitive test	Description	Outcome measure	Unit
*Domain: Attention and psychomotor speed*			
Motor screening task^a^ (CANTAB)	Colored crosses (“stimuli”) appear in various locations on the screen. The participant must select the cross as quickly and accurately as possible.	**1. Mean latency for a participant to correctly respond to the stimulus on screen during assessed trials**	milliseconds
		2. Total number of correct responses a participant made across all assessed trials	number
		3. Total number of incorrect responses a participant made across all assessed trials	number
Reaction time (CANTAB)	Participants must select a button on the screen. Circles are presented above the button and the participant is asked to react as soon as possible once a yellow dot appears in one of the circles by clicking the corresponding circle. The assessment entails two tasks: - Simple task: one circle above button - Five-choices task: five circles above button.	**1. Mean reaction time in** simple and **five-choices task**	milliseconds
		2. Mean movement time in simple and five-choices task	milliseconds
		3. Total error score in simple and five-choices task	number
		4. Inaccurate response error score in simple and five-choices task	number
		5. No response error score in simple and five-choices task	number
		6. Premature responses error score in simple and five-choices task	number
		7. Inaccurate location error score in five-choices task	number
Rapid visual information processing (CANTAB)	A white box is shown in the center of the screen where digits from 2 to 9 appear in a pseudo-random order at the rate of 100 digits per minute. Participants are requested to detect target sequences of digits (e.g., 2-4-6, 3-5-7, 4-6-8). When the participant sees the target sequence, they must respond by selecting the button in the center of the screen as quickly as possible.	**1. A prime: signal detection of a participant’s sensitivity to the target sequence regardless of response tendency, i.e. measure of how good the participant is at detecting target sequences**	-
		**2. Probability of hit: the number of target sequences during assessment blocks that were correctly responded to within the time allowed, divided by the number of target sequences during assessment blocks**	-
		**3. Probability of false alarm: the number of sequence presentations that were false alarms divided by the number of sequence presentations that were false alarms plus the number of sequence presentations that were correct rejections**	-
		4. Total hits: the total number of target sequences that were correctly responded to within the allowed time during assessment sequence blocks	number
		5. Total misses: the total number of target sequences that were not responded to within the allowed time during assessment sequence blocks	number
		**6. Mean response latency on trials were the participant responded correctly across all trials**	milliseconds
*Domain: Memory*			
Paired associates learning (CANTAB)	Boxes are displayed on the screen and are opened in a randomized order. One or more of them will contain a pattern. The patterns are then displayed in the middle of the screen, one at a time and the participant must select the box in which the pattern was originally located. The entire assessment contains multiple levels with an increasing number of patterns.	1. Errors: the total number of times a participant selected an incorrect box when attempting to recall a pattern location (expressed for the entire assessment and per level)	number
		**2. Adjusted errors: the total number of times a participant selected an incorrect box when attempting to recall a pattern location, plus an adjustment for the estimated number of errors they would have made on any assessment level, attempts and recalls they did not succeed on (expressed for the entire assessment and per level)**	number
		3. Attempts: the total number of attempts made by the participant during assessment levels (expressed for the entire assessment and per level)	number
		4. Mean errors to success: the mean number of attempts needed by a participant to successfully complete the level	number
		**5. First attempt memory score: the number of times a participant chose the correct box on their first attempt when recalling pattern locations**	number
		6. Number of patterns reached: the number of patterns presented to the participant on the last level they reached	number
Spatial working memory (CANTAB)	Several colored boxes are shown on the screen. The aim of this test is that, by selecting the boxes and using a process of elimination, the participant should find one yellow ‘token’ in each of several boxes and use them to fill up an empty column on the right-hand side of the screen. The entire assessment contains multiple levels with an increasing number of boxes.	**1. Between errors: the number of times the participant incorrectly revisits a box in which a token has previously been found (expressed for the entire assessment and per level)**	number
		2. Within errors: the number of times a participant revisits a box already shown to be empty during the same search (expressed for the entire assessment and per level)	number
		3. Double errors: the number of times a participant commits an error that is both a within error and a between error (expressed for the entire assessment and per level)	number
		4. Total errors: the total number of times a box is selected that is certain not to contain a token and therefore should not have been visited by the participant (expressed for the entire assessment and per level)	number
		**5. Strategy: the number of times a participant begins a new search pattern from the same box they started with previously**^b^	number
		6. Level reached: reports the level number that the participant reached but did not necessarily completed	number
Verbal recognition memory (CANTAB)	The participant is shown a list of words to remember. During the recognition phase, the participant is asked to say whether they remember seeing the word on the screen before. The word can be one of the originals, or a new word (distractor) which they have not yet seen before.	1. Free recall distinct stimuli: the total number of distinct words that are correctly recalled from the presentation phase	number
		2. Free recall novel words: the total number of novel words that were given by the participant which were not shown during the presentation phase	number
		3. Free recall perseverations: the number of times a participant repeats a word that was shown during the presentation phase	number
		**4. Immediate total correct: the total number of target words that the participant correctly recognizes plus the total number of distractor words that the participant correctly rejects**	number
		5. Immediate correct to stimuli: the total number of words that the participant correctly recognizes in the immediate recognition phase	number
		6. Immediate correct to distractors: the total number of times the participant correctly responds “no” to a distractor word in the immediate recognition phase	number
		7. Immediate incorrect to distractors: the total number of times the participant incorrectly responds “yes” to a distractor word in the immediate recognition phase	number
		**8. Delayed total correct: the total number of target words that the participant correctly recognizes in the delayed recognition phase, plus the total number of distractor words that the subject correctly rejects**	number
		9. Delayed correct to stimuli: the total number of words that the participant correctly recognizes in the delayed recognition phase	number
		10. Delayed correct to distractors: the total number of times the participant correctly responds “no” to a distractor word in the delayed recognition phase	number
		11. Delayed incorrect to distractors: the total number of times the participant incorrectly responds “yes” to a distractor word in the delayed recognition phase.	number
*Domain: executive function*			
Trail making test	The test consists out of two parts, A and B, and is performed with paper and pencil. During part A, the participant needs to connect 25 digits in ascending order (1 to 25) as fast as possible by drawing lines between the digits. During part B, the participants need to connect 13 digits in ascending order (1 to 13) and 12 letters (A to L) in alphabetical order, alternating digits, and letters (i.e., 1 – A – 2 – B – 3 – C, etc.) as fast as possible by drawing lines between the digits and letters.	**1. Time of completion part A and B**	seconds
		2. Amount of errors part A and B	number
		**3. Time of completion part B/part A**	-
		**4. Time of completion part B minus part A**	seconds

^a^the motor screening task will not be used for the calculation of the global cognitive score. This test is a screening task to make sure there are not sensorimotor deficits or lack of comprehension present

^b^strategy is also a measure of executive function

Cognitive function is selected as a primary outcome as the research on cognitive function and brain health in people with COPD is scarce [27, 28], and the pathogenesis of cognitive dysfunction is not fully understood. A global cognitive score constitutes the primary outcome, as recommended by the outcome of a recent Nature meta-analysis on the effects of exercise on cognition [65]. Current research on exercise prescription and treatment of cognitive dysfunction in people with COPD is still in the initial stages [27, 29, 37]. To our knowledge, no trial has compared the effect of different exercise intensities on global cognitive function in people with COPD.

The CANTAB is quick to set up and easy to administer, measuring sustained attention, psychomotor speed, episodic memory, working memory, and executive function. The cognitive test battery has been used in various populations but not yet in COPD. The separate cognitive domains have, however, previously been assessed in individuals with COPD following exercise training [27, 66]. The CANTAB will be administered in country-specific translations using the CANTAB Connect Research software on an Apple iPad in a quiet room. Automated voiceover and written instructions will be given by the software. The test battery takes around 60 min to complete, and an assessor will be nearby and available for any questions. The TMT is a widely used neuropsychological test tool to evaluate executive functions such as selective attention and cognitive flexibility. The test is administered into two parts, TMT-A and TMT-B (Table 6).

In addition to the global cognitive score, each separate test score will also be assessed as a secondary outcome (Table 6). The seven tests will be performed in the following order: motor screening task (not included in the calculation of the global cognitive score), spatial working memory, verbal recognition memory, TMT, reaction time, paired associates learning, and rapid visual information processing. Multiple outcome measures are available for each test, with outcomes that are recommended by CANTAB and previously used in similar research [67–69] highlighted in bold in Table 6. Which test-specific outcomes that will be included in the global cognitive score determination will be detailed in the statistical analysis plan (SAP).

#### Cardiorespiratory fitness

Cardiorespiratory fitness will be assessed as the maximum oxygen uptake (VO_2_peak (ml O_2_/min/kg)) during a standardized ramp-protocol CPET on a cycle ergometer [70].

This measure is selected as a primary outcome as it is clinically relevant and a physiological adaptation in cardiorespiratory fitness following an exercise intervention period is a vital objective in international COPD guidelines [9]. The CPET provides an assessment of the integrative exercise responses involving the pulmonary, cardiovascular, and skeletal muscle system, provides quantification of the level of impairment, and measures the effects of an intervention [9, 70]. It is also of importance to determine whether the interventions were successful at changing the cardiorespiratory fitness, which might mediate changes in cognitive function.

The CPET will start with a 3-min resting phase before a 3-min unloaded (or lowest wattage possible) warm-up phase. Next, the ramp-phase will start at 10 to 20 W and the loading will increase with 5 to 20 W per minute (automatically and continuously, i.e., ramp protocol). In Sweden, the choice of ramp protocol is guided by normative values for expected peak work rate. The rate of increase in work rate will be based on sex, age, height, disease severity, physical activity, and estimated exercise capacity based on a consultation with the participant. In Belgium, the rate of increase in work rate will be set at 5 to 10 W per minute for persons with COPD and 10 to 20 W per minute for healthy persons dependent on their self-reported physical activity. The aim is to have a ramp phase of 8–12 min. Target pedalling cadence will be 50–70 RPM. After the end of the ramp phase, there is a 3-min recovery phase with unloaded (or lowest wattage possible) pedalling at a reduced cadence of 30–50 RPM.

All cycling tests, including the CPET, the BCST, and the constant work-rate test (CWRT) (see secondary outcomes below), will be performed on a commercially available RPM-independent electromagnetically braked cycle ergometer. The cycle-ergometers will be calibrated according to manufacturer’s specifications. Cycle ergometers will be adjustable (handlebar and seat), be able to increase the work rate automatically and continuously, and allow for a low work rate (close to zero W) during unloaded pedalling. During all cycle test assessments, indirect calorimetry will be performed to analyse expired and inspired gas volumes (O_2_, CO_2_) and airflow parameters (ventilation, L/min). Metabolic carts using the breath-by-breath methodology will be used. The systems will be calibrated before each test according to manufacturer’s guidelines. Blood pressure will be manually or automatically measured with a sphygmomanometer. Electrocardiography (ECG) is collected with a standard 12-lead ECG. Heart rate (in beats per minute) is measured with ECG or chest strap and oxygen saturation with pulse oximetry. See Table S1-S2 in the online supplement additional file 4, for an overview of the equipment and their manufacturers at both trial sites.

#### Quadriceps power

Quadriceps power will be assessed as peak power (PP, in W) during a seated leg extension performed on a computerized dynamometer (Biodex System 3 or 4, Biodex Medical Systems Inc., Shirley, USA).

Quadriceps power is chosen as a primary outcome because it is the muscle property that seems to be most closely related to functional capacity [71] in healthy older adults and in people with COPD. Muscle power is also linked to daily physical activity [72]. Notably, studies comparing high-velocity interventions (e.g., HIIT) vs. conventional training programs (e.g., MICT) are highly warranted [73].

For the muscle power, muscle strength, and muscle endurance (secondary outcomes), measurements will be performed unilaterally on the dominant leg during the same trial visit. The muscle power assessment will be first, followed by muscle strength, and lastly, muscle endurance. Resting time between the muscle function tests is 3–5 min. The dominant leg will be defined as the one that the participant would kick a football with. Participants will be seated on a backward-inclined chair. A strap will be applied across the thigh that will be used in the test. To minimize movement, hips and shoulders will be stabilized with safety belts. The rotational axis of the dynamometer will be aligned with the transversal knee–joint axis and connected to the point of force application at the distal end of the tibia, approximately 5 cm above the lateral malleolus. Range of motion (ROM) will be individualized using a string placed at the end of ROM minus 5°.

For the muscle power assessment, participants will be instructed to extend their leg as fast and as hard as possible against a fixed resistance of 0.5 Nm and then passively return the leg to the starting position. A warm-up with ten contractions against 0.5 Nm, with increasing effort from the participant, will precede the assessment. After 2 min of rest, two explosive contractions will be performed in which PP and peak velocity (PV, in ˚/s) will be determined. At least 1 min of rest will be given in between the first and second contractions. Peak velocity will be used as a secondary outcome. These measurements have shown excellent relative test–retest reliability (intra-class correlation > 0.96) and with 95% limits of agreement for PV of 0 to 9% and for PP 1 to 20% [74].

### Secondary outcomes

#### Exercise tolerance

Exercise tolerance will be assessed as test duration (mm:ss) of a CWRT on a cycle ergometer to voluntarily exhaustion [75, 76]. End-exercise and iso-time parameters, e.g., VO_2_ (ml/min/kg), ventilation (L/min) dyspnoea and RPE, will also be assessed and presented as absolute value and as percentage of peak value obtained during CPET. Capillary blood will be collected before, during (every fifth minute), and 2 min after the CWRT to investigate lactate concentration (mmol/L). As such, we will investigate the metabolic demand and metabolic flexibility during CWRT before and after the interventions, as well as compare people with COPD to healthy controls in this manner. Changes in operational lung volumes will be obtained by performing inspiratory capacity manoeuvres every minute during rest, at the end of warm-up, every other minute during the test, and at the end of cool-down [77].

After a 4-min resting phase and a 3-min unloaded warm-up phase, participants will cycle at a constant workload corresponding to 75% of MAP for COPD and 85% of MAP for HC with a self-selected pedalling cadence of 50–70 RPM. Participants will be instructed to exercise for as long as possible. The test will stop at symptom limitation or failure to maintain a cadence of ≥ 50 RPM for 10 s. After the test phase, there is a 3-min recovery phase with unloaded (or lowest wattage possible) pedalling at a reduced cadence of 30–50 RPM. For participants with a limited cardiorespiratory fitness, defined as a CPET duration shorter than 6 min, the warm-up phase is reduced to 2 min.

If participants cycle shorter than 2.5, or longer than 15 min at baseline, the test will be performed again at a ± 5–10% or ± 5–10 W change in intensity another day. For participants reaching a MAP ≥ 100 W, a 5–10% change in intensity will be used. For participants with a MAP < 100 W, a 5–10 W change in intensity will be used. For the post-intervention testing, there will be an upper time limit of 60 min. Standardized encouragement will be given during the test.

#### Anaerobic exercise capacity

Anaerobic exercise capacity will be assessed as intensity (absolute workload (watts), relative (% of MAP)) at the final stage during a BCST and as estimated MPO_6_. End-exercise and iso-time parameters, e.g., VO_2_ (ml/min/kg), ventilation (L/min), dyspnoea and RPE, will also be assessed, and presented as absolute value and as percentage of peak value obtained during CPET.

The BCST [48] is a submaximal cycle ergometer test that is used to estimate maximum mean power output for 30 s (MPO_30_) without an all-out effort. The BCST consists of 30-s efforts with increasing intensity, interspersed with 30-s passive recovery between each effort. With a target pedalling cadence of 80–90 RPM, participants cycle until they score an RPE of ≥ 17, or when they cannot keep the target cadence (< 75 RPM for 5 s). Would a participant continue until they cannot keep the target cadence or stop due to exhaustion at baseline testing, a RPE of 17 will not be used as a stopping criterion in follow-up testing. If a participant cannot maintain a cadence of 80–90 RPM during a familiarization bout, a lower (60–70) RPM will be used. In this case, the target cadence stopping criteria will be < 55 RPM for 5 s. Standardized encouragement will be given during the test.

We have tailored the test to our population of interest by modifying the initial load (15–50 W) and rate of increase (15–50 W) compared to the original BCST performed in young healthy adults [48]. The initial load and rate of increase is set depending on the participant’s previously determined MAP, where the aim is to reach a RPE of 17 after four to six stages. Incomplete 30-s stages with ≥ 10-s duration will be considered for the MPO_6_ calculation, by adding a certain load corresponding to the percentage of the completed stage (i.e., 15 s completed at 50 W increase will add 25 W to the last completed stage). The MPO_6_ is estimated by multiplying the power during the last completed stage by 1.75 as previously described [15] and successfully used in our research group in people with COPD [17, 78, 79].

The BCST has been shown to be feasible and effective in older adults to estimate the MPO_6_ and has in our research group also been used successfully in people with COPD as a test and to prescribe supramaximal HIIT. In our feasibility trial, the intensity during the last BCST stage was, on average, 150% of MAP (*n* = 32) [78, 79]. Subsequently, the MPO_6_ was on average 260% of MAP.

#### Magnetic resonance imaging-derived brain measures

MRI outcomes include grey matter volume (mm^3^), area (mm^2^), thickness (mm), and primarily hippocampus and cortical thickness [37, 80, 81] and cerebrovascular indicators such as white matter lesion volumes, lacunes, perivascular space dilation, microbleeds, and cerebral blood flow (mL/100 g/min). Additionally, a functional MRI (fMRI) will be performed using the face–name association task.

The face–name task is designed to address verbal and non-verbal memory functions relatively equally as face–name associations have been shown to rely on left and right mesial temporal lobes and elicit bilateral hippocampal activations in healthy participants. Of relevance in COPD considering abnormal static and dynamic local–neural activities in the parahippocampal and hippocampal cortex found in individuals with COPD and its relationship with poor lung function and semantic memory impairments [82], as well as observed hippocampal volume reductions in COPD compared to healthy adults [80].

The MRI scans are performed in a 3 T Discovery MR 750 scanner (General Electric, WI, US) at the Umeå centre and a Ingenia Elition X 3.0T (Philips, Amsterdam, The Netherlands) at Jessa Ziekenhuis, Hasselt centre. T1-weighted images are obtained and processed with the Freesurfer pipeline to extract volumes of grey matter, white matter, and lateral ventricle size. Subcortical grey segmentations and cortical parcellations are achieved to define regions of interest (e.g., hippocampus or cortex). T2-weighted, fluid-attenuated inversion recovery (FLAIR), and susceptibility weighted images are collected to assess perivascular spaces, lacunes, white matter lesions, and microbleeds. More specifically, white matter lesions are segmented and quantified from FLAIR images with the lesion growth algorithm implemented in the lesion segmentation toolbox in the open source statistical parametric modelling software (SPM). Perivascular space dilation and lacunes are inspected from T2-weighted and FLAIR images, and microbleeds are inspected from susceptibility images. Cerebral blood flow is measured with 3D pseudo-continuous arterial spin labeling with background suppression and spiral readout. Mean grey matter perfusion (ml/100g/minute) is computed for Freesurfer-segmented regions of interest as the average of the individual perfusion estimates weighted by volume. The MRI scan procedures take 1 h.

#### Neuroinflammation

In vivo neuroinflammation is measured as uptake rate (ki) of ^11^C-deprenyl in the brain assessed with PET/CT (General Electric, WI, USA) under resting-state conditions after intravenous injection of 314 MBq of ^11^C-Deprenyl. The PET/CT scan is performed at baseline (COPD and healthy controls) and at 24 months (COPD).

Neuroinflammation is quantified to assess how muscle-related processes are related to inflammation within the brain and whether it can be reduced via physical exercise [83–85]. The radioligand ^11^C-deprenyl binds to monoamine oxidase B (MAO-B), a protein that is upregulated when glial cells are activated, such as during inflammation [86]. Uptake rate of ^11^C-deprenyl in the brain is modeled with Patlak analyses, where higher uptake is representative of higher levels of inflammation. Following Swedish National guidelines, the total radiation dose received will not exceed five mSv. The PET/CT scan takes 1 h.

#### Mild cognitive impairment

Baseline cognitive function and prevalence of mild cognitive impairment will be assessed using the Montreal Cognitive Assessment (MoCA) test (0–30 points). The MoCA is often used in COPD research [87, 88] and evaluates multiple domains of cognitive function [89]. The MoCA tests eight cognitive domains and takes 10–15 min to administer. The MoCA will be performed at baseline and 24 months.

#### Blood sample-derived outcomes

Fasted venous blood samples will be collected in a resting state to investigate the circulating levels of neurotrophic factors, exerkines, markers of systemic inflammation and blood lipid profile (plasma or serum concentration, and epigenetic modifications in whole blood). Resting lactate will be measured in capillary blood in a non-fasted state.

- *Neurotrophic factors and exerkines related to muscle-brain crosstalk****.*** Main analytes include BDNF, serum insulin-like growth factor-1 (IGF-1), lactate, vascular endothelial growth factor (VEGF), irisin, cathepsin B, clusterin, KYN, and KYNA. Measured in pq/L or as appropriate, except for lactate which is measured in mmol/L.

BDNF [31–34] is specifically targeted since it is believed to at least in part mediate potential benefits of exercise on brain function due to its ability to stimulate growth and differentiation of new neurons and synapses [26].

- *Markers of systemic inflammation.* Markers of systemic inflammation, including but not limited to highly sensitive C-reactive protein (hs-CRP [mg/L]), fibrinogen (g/L), interleukin-6 (IL-6), IL-8, and tumour necrosis factor alpha (TNF-α), are measured in pq/L.
- *Markers of cardiometabolic health and blood lipid profile.* Total cholesterol, low-density lipoprotein (LDL), high-density lipoprotein (HDL), non-HDL, triglycerides, and glucose measured in mmol/L, insulin (mIU/L), and haemoglobin A1c (HbA1c, [%]). Glucose and insulin levels will be used to calculate the Homeostatic Model Assessment for Insulin Resistance (HOMA-IR) [90].
- *Complete blood count with differential.* A differential blood count will be performed to primarily analyse the total white blood cell count and eosinophil count in whole blood (count × 10^9^/L), which is related to inflammation and exacerbations in people with COPD [91].
- *Epigenetic modifications and genotyping.* Genome-wide DNA methylation (differentially methylated cytosines) will be analysed via the EPICarray technology following manufacturer’s guidelines at H42, a company specialized in epigenetic analysis, based at the Faculty of Pharmaceutical Sciences of Ghent University (Belgium). We will also do a targeted genotyping approach for *APOE*, known to be related to cognitive function [92].
- *Blood sample procedures*. Fasted venous blood samples will be collected in a seated position with a 21–22-gauge needle from the antecubital fossa or back of the hand. Blood samples will be collected between 7.45 and 10.30 AM after a 15-min seated rest. Per every time point, the blood is collected into three K_2_EDTA tubes, one serum-separator tube (SST), one lithium–heparin tubes, and one citrate tube (BD Vacutainer, Franklin Lakes, NJ, USA). A total of approximately 30 mL of blood will be collected per time point. Tubes will be immediately homogenized by inverting five to ten times, as appropriate depending on type of tube. One EDTA tube, and the citrate plasma tube, will be immediately sent for analysis (complete blood count and fibrinogen, respectively) and not undergo cryopreservation. The remaining tubes will be sent to the local biobank, and after 30 min at room temperature to allow for clotting in the SST, the tubes will be centrifuged at 1500 × *g* for 10 min (whole blood samples will be aliquoted from EDTA tube before centrifugation). Samples will then be aliquoted into serum, plasma (EDTA and lithium-heparin), erythrocytes (EDTA), and buffy coat (EDTA). Aliquots of 200–500 µL will be stored in − 80°C within 1 h of blood collection.

Blood samples will be stored at each centre’s biobank until analysis. To maintain sample stability in cryopreserved specimens, unique aliquots will be used for every analysis. Thus, analysed samples will only undergo one freeze–thaw cycle. Blood samples will be analysed either using commercially available kits following manufacturer’s guidelines or at the Clinical Chemistry facilities at NUS (Umeå, Sweden) or at ZOL (Genk, Belgium) using standard routines. Intra- and inter-assay coefficients of variation will be reported along with the results.

Notably, the specific blood markers to be analysed might be updated in response to new methods, challenges encountered during the trial, funding availabilities, or new evidence during the period of the trial.

#### Quadriceps strength and endurance

Muscle strength will be determined as the one-repetition maximum (1-RM) defined as the maximum torque (Nm) able to be lifted through the full range of motion (ROM) during seated leg extension in the dominant leg. Muscle endurance will be assessed as the maximum number of repetitions able to be lifted with a good technique through the full ROM at an intensity corresponding to 45% of 1-RM [93].

Muscle strength assessment will start with a warm-up consisting of ten submaximal contractions at a low and individualized resistance. Next, the strength test will consist of several 1-RM trials with progressive resistance increments until the maximum resistance that can be lifted throughout the ROM has been reached. A 3–5-min rest will be used between trials.

Muscle endurance will be assessed after muscle strength, and a warm-up of ten submaximal repetitions at 22.5% of the 1-RM will be performed. During the muscle endurance assessment, the participants will be instructed to do as many repetitions as possible using a resistance of 45% of the 1-RM. The pace will be standardized and externally set by a metronome at 60 beats per minute. Neutral feedback will be given concerning ROM and pace.

#### Functional performance

Functional performance will be assessed as the time needed to complete the five time sit to stand test (5-STS) [94] and calculated average power (W) during ascent of a ten-step flight of stairs during the stair climbing power test (SCPT).

The 5-STS will be performed in line with recommendations [94]. Participants will be instructed to transfer from a seated to a standing position and back five times as fast as possible. The time needed to complete the task is recorded to the nearest 0.01 s. Mean functional muscle power will be calculated from the 5-STS time using the following equation: power-5STS (W) = (body mass × 0.9 × *g* × (height × 0.5 − chair height)) / (time 5-STS × 0.1) where *g* is the acceleration of gravity (9.81 m/s^2^) and height is expressed in metres [95]. A standardized chair height of 45 cm will be used, and feet placement measured and replicated in follow-up tests.

In the SCPT, participants will be instructed to perform two trials where they ascend a well-lit ten-step flight of stairs as fast and safely as they can, using the handrail if necessary for safety purposes at the command ‘Ready, set, go’. The start position of the SCPT is to stand at the base of the ten-step flight of stairs. The time needed to complete the task is recorded to the nearest 0.01 s and starts after the command ‘go’ once the subject begins to move and ends when both feet reach the top step. The assessor will be located at the middle of the stairs and if needed, staying close to the participant during the trial. Stair climb time and total vertical height of stairs are used to calculate velocity (distance/time; *v* = *d*/*t*). Body mass and acceleration due to gravity (9.81 m/s^2^) are used to calculate force (mass × gravity; *F* = *m* × *a*). Power is the product of force and velocity (force × velocity; *P* = *F* × *v*). The mean of the two trials is recorded and recovery time between trials should be at least 2 min.

#### Cardiovascular function

- Modulation of autonomic cardiac function and resting heart rate: autonomic cardiac function is measured via resting state heart rate variability (HRV). Resting heart rate (RHR) will also be obtained during the HRV measurement. After a 10-min seated rest, HRV and RHR will be measured during a 5-min period with a Polar H10 chest strap with a 1000 Hz sampling frequency, according to guidelines [96, 97]. Participants will be sitting in a relaxed position, breathing spontaneously for the duration of the recording. Data will be processed and analysed using the latest version of the Kubios HRV scientific software [98] (Kubios Oy, Kuopio, Finland). Outcomes in the time domain (e.g., root of the mean square of successive R–R interval differences (RMSSD, in ms), SD of normal-to-normal R–R intervals (SDNN, in ms), number of successive R–R interval pairs that differ more than 50 ms (NN50, in %), and in the frequency domain (total, low- and high-frequency power (LF, HF) and LF:HF ratio) will be analysed.
- Blood pressure: resting blood pressure (systolic and diastolic; mmHg) will be obtained after 15-min seated rest using a calibrated automatic sphygmomanometer (Welch Allyn 3400, Skaneateles Falls, New York, USA). Two measurements will be performed with 1-min rest in between measurements. The second measurement will be used for analysis.

#### Muscle sample-derived outcomes

Muscle biopsies from the middle part of the m. vastus lateralis will be collected to investigate muscle morphology, metabolic enzymes, mitochondrial biogenesis, angiogenesis, myokines and related compounds linked to muscle–brain crosstalk, and epigenetic modifications. Muscle biopsies are taken at baseline and 12 weeks. Target sample size is 75% of the total sample size in phase 1.

These parameters are to be analysed:

- Expression of slow and fast subtypes of contractile myosin heavy chain isoforms in fibres as previously described [99] (relative distribution in %, relative area of the muscle occupied by the different subtypes of fibres in %).
- Muscle fibre cross-sectional area [99] (of all fibres and per subtype, µm^2^).
- Muscle capillarization: capillaries per fibre (capillaries in close contact with fibre; number), capillaries per fibre area (ratio capillaries per fibre/fibre area), sharing factor (capillary-to-fibre ratio), and capillary density (number of capillaries/mm^2^) [100]. Capillaries per fibre and per fibre area will be calculated for all fibres and subtypes.
- Myofibre and mitochondrial disruption/abnormalities: staining of reduced nicotinamide adenine dinucleotide–tetrazolium reductase, succinate dehydrogenase, and cytochrome c oxidase (% of fibres and subtypes with abnormal staining pattern, fibre area in µm^2^ of fibres with abnormal staining pattern) [101].
- Activity and content of metabolic enzymes: Phosphofructokinase (PFK; glycolysis), lactate dehydrogenase (LDH; anaerobic glycolysis), citrate synthase (CS; Krebs cycle), succinate dehydrogenase (SDH; Krebs cycle), 3-hydroxyacyl Coenzyme A dehydrogenase (HADH; beta-oxidation), mitochondrial complex I-V (oxidative phosphorylation) [102] (mmol or µmol/min/mg or g, ratio, fold change or arbitrary units).
- Mitochondrial biogenesis: mRNA and protein level of PGC-1α and PGC-1β, transcription factor A mitochondrial (TFAM), nuclear respiratory factors (NRF1 and NRF2α), and peroxisome proliferator-activated receptor-α (PPAR-α) [102] (fold change or arbitrary units).
- Angiogenesis: mRNA and protein level of VEGF (fold change or arbitrary units).
- Myokines and related compounds linked to muscle–brain cross-talk [103]: mRNA and protein level of BDNF, fibronectin type III domain-containing protein 5 (FNDC5), and kynurenine aminotransferase (KAT1-4) [104] (fold change or arbitrary units).
- Epigenetic modifications: genome-wide DNA methylation (differentially methylated cytosines) will be analysed via the EPICarray technology as described above.

Notably, the parameters to be analysed might be updated in response to new methods, challenges encountered during the trial, funding availabilities, or new evidence during the period of the trial.

Muscle biopsies from the middle part of the m. vastus lateralis (dominant leg) will be collected under local anaesthesia via the Bergström needle technique by an experienced physician. The participant will be in supine resting position during obtainment of the muscle biopsy. An experienced researcher will perform the processing of muscle biopsies as follows: (1) transport of the muscle biopsy on sterile gauzes wetted by NaCl in a petri dish on ice; (2) fat tissue is cut off from the muscle biopsy and the muscle biopsy it padded dry; (3) a part of the muscle sample will be snap frozen in liquid nitrogen for enzyme activity analysis via spectrophotometry, mRNA and protein expression analysis via qPCR and western immunoblotting, respectively (Maastricht University, Maastricht, The Netherlands), and DNA methylation analysis via EPICarray technology (H42 and Ghent University, Ghent, Belgium); and (4) another part of the muscle biopsy will be attached via optimum cutting temperature compound to a mount and frozen in *melting* propane (cooled by liquid nitrogen) for 15–30 s for immunohistochemical and morphological analysis (Umeå University, Umeå, Sweden). Muscle samples will be stored at − 80 °C in the centre’s respective biobank until analysis.

#### Body composition

Body weight and body composition will be assessed at all timepoints. Body composition measures include body mass index, waist, and hip circumference (cm), waist/hip ratio, fat mass (kg and %), lean mass (kg and %), and lean mass index (kg/m^2^). Body composition will be measured using bioelectrical impedance (Bodystat 1500, Bodystat Ltd., Isle of Man) following manufacturer’s procedures. Body weight will be measured with a calibrated weight scale.

#### Impact of COPD on daily life

Impact of COPD on daily life will be determined using the CAT [105]. The CAT will only be obtained in the COPD group. The CAT is an eight-item questionnaire that assesses the impact of COPD on self-reported health status and symptoms [105]. Each item is scored from 0 to 5 points where 0 indicates no impact or symptoms, and 5 indicates the worst possible impact or symptoms. Subsequently, scores are summed to a total CAT score of 0–40 points. The CAT takes 3–5 min to complete. The CAT will be measured at baseline, 12 weeks, and at months 6, 9, 12, 15, 18, 21, and the 24-month follow-up.

#### Disease-specific quality of life

For assessment of disease-specific quality of life, the CRQ [106] (SAS version; Self-Administered Standardized) will be used. The CRQ will only be obtained in the COPD group. The CRQ measures the impact of chronic respiratory disease on HRQoL and covers four key domains of HRQoL, including: dyspnoea, fatigue, emotional function, and mastery. Each item is scored on a 7-point Likert scale; scores within each domain are summated for a total score per domain. Research has shown that the CRQ is a valid and responsive test of HRQoL and correlates well with clinical status [107, 108]. The CRQ will be measured at baseline, 12 weeks, and at months 6, 9, 12, 15, 18, 21, and the 24-month follow-up.

#### Health-related quality of life (HRQoL)

Quality of life is assessed with the EQ-5D-5L. It assesses an individual’s HRQoL in five dimensions: mobility, self-care, usual activities, pain/discomfort, and anxiety/depression [109]. Each dimension has five response levels, ranging from level 1 (no problems) to level 5 (extreme problems). By combining one level from each dimension, a health state ranging from 11111 (the best health state) to 55555 (the worst health state) is defined and converted into an index score using a scoring algorithm. The EQ-5D-5L is a valid and responsive measure of HRQoL, also in COPD [110]. The instrument also includes a visual analogue scale, which provides a single global rating of self-perceived health. The scale is scored from 0 (‘the worst…’) to 100 (‘the best health you can imagine’). The EQ-5D-5L takes approximately 5 min to complete.

#### Depression and anxiety

The Hospital Anxiety and Depression Scale (HADS) [111] will be used to assess states of depression and anxiety. The HADS is a reliable 14-item questionnaire regarding feeling of depression (seven items) and anxiety (seven items) in the past 7 days. The items are rated on a 0 (no impairment) to 3 (severe impairment) point severity scale, with a maximum score of 21 for depression and anxiety, respectively. The HADS takes approximately five to ten minutes to complete.

#### Self-perceived change

Self-perceived change in outcomes will be measured via the Global Rate of Change [112] scale with the purpose to quantify the extent to which a participant perceive they have improved or deteriorated over time in the outcome. Participants will provide an answer via a 11-point Likert scale (− 5 = ‘Much worse’; 0 = ‘Unchanged’; 5 = ‘Much better’). The question about self-perceived change will be posed after the following outcome assessments at follow-ups: cardiorespiratory fitness (CPET), anaerobic capacity (BCST), quadriceps power, quadriceps strength, quadriceps endurance, global cognitive function, exercise tolerance (CWRT), 5-STS and SCPT. In addition, they will also rate their perceived change in overall health.

#### Number of responders

Total amount of responders, defined as a response over the known minimal detectable change or minimal important difference for included tests, will be determined and compared between supramaximal HIIT and MICT.

#### Feasibility of interventions

Feasibility of the training programs will be assessed and evaluated between groups using both quantitative and qualitative measurement methods:

- Completion rate is determined by the total number of participants still performing the exercise training intervention at 12-week and 24-month follow-up. Reasons for non-completion, i.e., dropouts, are also obtained.
- Attendance rate is determined by the total number of attended sessions divided by total number of sessions prescribed, presented as a percentage. Reasons for non-attendance are also obtained.
- Adherence to exercise duration is determined as the adherence to the predefined intervals/minutes of the exercise training. Reasons for non-adherence are also obtained.
- Adherence to exercise intensity is determined as the adherence to the prescribed intensity of the exercise training. Reasons for non-adherence are also obtained. Exercise intensity (absolute workload (W), relative intensity (% of MAP, % of MPO_6_), the level of dyspnoea and leg fatigue (0–10, arbitrary units) on the [47] CR10 scale [47], and perceived exertion (6–20, arbitrary units) on the [47] RPE scale [60]) measured throughout the intervention will be reported. In addition, approximately 5 min after each exercise session, a session RPE [113] will also be collected using the [47] CR10 scale.
- Attendance rate for resistance training is determined as the self-reported total number of attended sessions divided by total number of sessions prescribed, presented as a percentage.
- Adherence to resistance training is determined as the self-reported adherence to the predefined number of exercises and number of sets.
- Exercise fidelity is determined as the incidence of exercise sessions requiring modifications, defined as any deviation from the prescribed exercise such as change in duration, intensity, or cadence.
- Occurrence and assessment of the severity of adverse events will be documented. Severity of adverse events will be rated into four different categories: (1) minor and temporary, (2) serious symptoms (potential risk of severe injury or life-threatening, (3) manifest injury or disease, and (4) death, as previously described [114]. An adverse event rate will be calculated for each participant as the total number of sessions during which any adverse events occurred divided by the total number of attended sessions.
- Participant satisfaction with the performed exercise training (supramaximal HIIT / MICT) will be recorded by adaptation of an existing patient satisfaction questionnaire previously used for cycling exercises in COPD [115] (see Tables S3 and S4, additional file 4).

Feasibility will also be determined, in those with COPD, by exploring the participants’ experiences and perceptions of participating in the exercise program by individual face-to-face or telephone interviews after both Phase 1 and Phase 2. A semi-structured interview guide will be used with questions about positive and negative feelings during or after the training, perceptions of participating in the exercise groups, the exercise format (supramaximal HIIT and MICT), perceived effects of the intervention, and if the program was experienced as doable and acceptable. We will also ask about their motivation for doing the intervention and thought about their future exercise. In the interview after phase 2, experiences presented in the first interview will also be followed up.

A sub-sample of up to 15 participants from each group (supramaximal HIIT and MICT) will be included in the interviews after phase 1. Participants with a variation in age, sex, symptoms (baseline CAT), and perceived effects of the intervention will be included for a varied sample. Both those who completed the intervention, and those who interrupted the intervention during either phase 1 or phase 2 will be invited. Everyone who completed an interview after phase 1 will be invited for the interview after phase 2, except for those who interrupted the intervention in phase 1 and did not enter phase 2, would there be any.

Qualitative content analysis [116] will be used for analysis in phase 1, while pattern-oriented longitudinal analysis [117] will be used to longitudinally analyse variations in change over time including both interviews. The COREQ checklist [45] will be used to ensure trustworthiness of the trial.

#### Hospitalisations

Number of hospitalisations per participant. Time, cause, length of stay, and emergency room visits (with no hospitalization afterwards) and their cause will also be extracted. Hospitalizations and emergency room visits will be classified as respiratory or non-respiratory. Data on hospitalizations will be extracted from the participant’s medical record and, when available, cross-checked with the participant’s diary (an outcome measure only in phase 2).

#### Exacerbations

Number of COPD exacerbations per participant (COPD only) during the trial period will be collected (an outcome measure only in phase 2).

#### Mortality

Number of deaths will be extracted from the participant’s medical record. Time and cause of each death will also be extracted (an outcome measure only in phase 2).

### Sample size

Sample size calculations have been made to ensure that we have a sufficient sample size to detect difference in change in all three primary outcomes for both Phase 1 and 2. For these a priori power calculations, the sample size has been determined assuming a two-sided alpha level of 0.05 and 80% power.

### Phase 1

#### Cognitive function

Assuming an SD of 0.4, a total sample size of 60 individuals (30 per group) is needed to detect a relevant difference of 0.27 in a global cognition z-score measured using a cognitive test battery [118]. A change in *z*-score of 0.27 is clinically relevant associated with increased all-cause, respiratory, and cardiovascular mortality. To account for a drop-out rate of approximately 15% at 3 months, a total of 70 participants is necessary.

#### Muscle power and cardiorespiratory fitness

A sample size of 70 individuals (*n* = 35 per group) is also sufficient to detect a difference of 19.4% (SD 26.8%) [74] in quadriceps power (*n* = 30 per group) and to detect a relevant difference in maximal oxygen consumption (VO_2_max) of 3.5 mL/kg/min (SD 4.6 mL/kg/min) [119] (*n* = 26 per group) measured during a CPET. A difference of 3.5 mL/kg/min is associated with a 15% decrease in risk for cardiovascular disease [120]. No clinically relevant difference exists for muscle power; however, a difference of 19.4% is the minimal detectable change (MDC) from repeated testing of quadriceps peak power in older adults, as calculated by MDC = 1.96 × √2 × standard error of measurement (SEM) [74].

### Phase 2

In total, 138 individuals with COPD will be included in phase 2 (supramaximal HIT (*n* = 46), MICT (*n* = 46), and usual care (*n* = 46)). The sample size is estimated to account for an up to 30% additional drop-out rate at 24 months, in line with previous literature [121–123]. See phase 1 for complete details on the main sample size calculation. Additionally, as reported above in phase 1, the SD of 0.4 in the cognitive test battery is estimated from a trial in healthy adults comparing low to higher-intensity exercises [118] and not in patients with COPD. In phase 1, this uncertainty will be addressed with the increase in sample size from 70 to 92. However, for phase 2, we will instead perform an interim blinded sample re-estimation after half of the participants have performed baseline and 24-month follow-up, using recommended procedures for non-comparative data [124, 125], and adjust the sample size accordingly. These adaptations are recommended when there is any uncertainty in the SD. Importantly, using non-comparative data does not inflate the type I error probability or induce any bias in the treatment effect estimate (as it would if comparative data had been used) [124, 125].

### Multicentre trial and covariates

The specific trial design is a stratified multicentre trial with individual randomization at each involved centre. Using this design results in a gain in power [126], not a loss in power typical of randomized cluster trials where observations on individuals in the same cluster tend to be correlated. Therefore, no increase in the sample size is necessary even though we collect data at two research centres. In contrast, the sample size could be reduced for a given power [126]. Furthermore, in the statistical analysis (described below), we will adjust the analysis for the predictive covariates age, sex, VO_2_peak, and centre—the latter as appropriate when stratifying the randomization. Adjusting for covariates in the statistical analysis is also associated with a gain in statistical power, or equivalently, a reduced sample size requirement [127–129]. Yet, we have taken a conservative approach and have not accounted for this when estimating the required sample size due to the complex assumptions the calculations require, leading to uncertainty of how much reduction in the required sample size can be expected.

### Allocation, randomization, and masking

In phase 1, participants will be randomized to either interventional arm or control arm with a 1:1 allocation ratio, using a computer-generated (Sealed Envelope Ltd., London, UK) blocked randomization schedule with varying block sizes of two or four, stratified by sex (male, female) and centre with equal allocation to all trial arms. For phase 2, only the participants with COPD will continue the maintenance program in their allocated group.

Group allocation will be performed after participants have been recruited into the trial; baseline assessments have been completed and eligibility confirmed. The allocation sequence will be kept in an opaque, tamper-proof sealed, and stapled envelope that will be concealed until the end of the outcome assessment. By using aluminium foil inside the envelope, it will be impermeable to intense light. A delegated person independent from enrolment, assessments, and training will generate the allocation sequence, enrol participants, and assign participants to interventions.

Outcome assessors and data analysts will be masked to group allocation. Participants will be instructed not to reveal their group allocation to the outcome assessor during interim-, 12-week, and 24-month trial assessments. In case of failure of keeping the outcome assessor masked, a second outcome assessor will be available. Given the nature of the interventions, neither participants nor instructors of the training sessions can be masked during the intervention. Hypotheses of the trial will not be revealed to the participants during their participation.

### Data management

The REDCap (Research Electronic Data Capture) Survey, a secure, Health Insurance Portability and Accountability Act compliant, web-based survey application will be used as an eCRF. The eCRF system has been set up by the ICT Services and System Development at Umeå University. The data is stored at local servers and is backed up daily. In the eCRF, quality control checks, such as validation of data type and expected data range, is used. Notifications and prompts are used if data is missing, outside of expected range or indicate participant eligibility. The majority of trial data is directly entered into the eCRF during and after trial visits, using a computer or tablet. Some data, such as ratings of exertion during exercise, will be collected on paper CRFs and later entered into the eCRF.

Other electronic data will also be generated outside of REDCap. This includes data files generated by metabolic carts (CPET, BCST, CWRT), lung function assessments, and blood and muscle sample data. These data will be stored on local databases designated for research data.

All data will be pseudonymized. Consenting participants will be assigned a unique sequential participant identification code. While this ID will be linked to personal data, only the ID code will be used during data collection and analysis to protect participants’ integrity. The participants’ identities will only be known by the research group at the respective centre and the PI. It will not be possible to identify specific individuals in the trial.

All hard-copy records with participant information, such as informed consent forms, will be stored separately in locked file cabinets in areas with limited access and separate from trial records identified by ID code at the local research centres, respectively. All personal data will be processed in accordance with the EU General Data Protection Regulation (GDPR).

### Statistical methods

The change-from-baseline analyses will be performed after the last participant has completed all assessments of phase 1 and phase 2, respectively, after monitoring and data quality checks. Analyses will be performed blinded by the investigators supported by a biostatistician. All statistical tests will be conducted as two-sided, with the alpha level set at 0.05.

Baseline variables and descriptive statistics with continuous data will be presented with mean (SD) or median (IQR) as appropriate. Continuous data with normative values (for example, VO_2_peak and lung function values) will also be presented as % predicted value. Ordinal variables will be presented as median (IQR) or as *n* (%), and categorical variables will be presented as *n* (%).

A separate detailed SAP with further details about the statistical analyses including the method to handle family-wise error rate for the multiple primary outcomes will be published and finalized before the database is locked and randomization codes unblinded. In line with recommendations, it increases transparency and replicability and might be updated in response to new methods, challenges encountered during the trial, funding availabilities, and emerging evidence during the period of the trial [130, 131].

#### Statistical methods—outcomes

For primary and secondary outcomes with continuous data, treatment effect will be estimated by mean change-from-baseline (delta-value (Δ)) with 95% confidence intervals and the effect size (ES). The between-group effects will be analysed using analysis of covariance (ANCOVA). In the ANCOVA, baseline values, age, sex, centre, and VO_2_peak will be used as covariates. In phase 1, the primary analyses will be one model for the COPD group and one model for HC. In addition, to investigate any differential effects to the interventions between COPD and HC, a two-way ANCOVA including the Group (COPD/HC) × Intervention (supramaximal HIIT/MICT) interaction will be performed. In phase 2, there will be one model with the three arms (supramaximal HIIT, MICT, and usual care). Additional details will be provided in the SAP.

The assumptions of linearity, normality of residuals, and homogenous variance will be checked using residual plots, normal quantile plots, and histograms. Violations of linearity and normality assumptions will be dealt with by transformations of the dependent variable or non-linear modelling of the independent variables. Outliers will not be removed unless there is a methodological reason for it.

Feasibility of intervention outcomes and number of responders will be described with descriptive statistics and analysed with between group (HIIT/MICT) comparisons in both the COPD (phase 1 and 2) and HC group (phase 1).

In phase 1, hospitalization and exacerbations during the intervention will be presented descriptively. In phase 2, mortality, hospitalizations, and exacerbations will be presented with descriptive statistics and analysed using Kaplan–Meier curves and Cox proportional hazard models.

In phase 2, pre-specified longitudinal mediation analyses will be performed on the entire MRI and PET/CT sample between changes in VO_2_peak, neurodegenerative measures, such as cognitive function and hippocampal volume and inflammation levels between baseline and 24 months. In these models, we will determine how inflammation mediates the effect of exercise training on neurodegenerative measures, including global cognitive function, hippocampal volume, and grey matter in the posterior, anterior, and midcingulate cingulate cortex, respectively, using a set of linear regression and maximum likelihood estimations [132]. Additionally, we will test whether the exercise training intensity (supramaximal HIT vs. MICT) has a different mediating effect. Additional details will be provided in the SAP.

#### Statistical methods—additional analyses

Pairwise comparisons between COPD and healthy controls will be conducted at baseline to investigate if matching has been successful, as well as to compare the cohorts using recommended methods for parametric and non-parametric data.

#### Statistical methods—analysis population, missing data, and sensitivity analyses

In line with recommendations [43], both adjusted and unadjusted analyses will be presented. Analyses will employ the intention-to-treat principle when applicable, meaning that all participants randomized, whether they receive their allocated intervention or withdrew from the trial, will be included in the analysis. Analyses will be performed using multiple imputation by chained equations (MICE) for missing data. Possible violation of missing at random assumption will be investigated through a sensitivity analysis, exploring plausible scenarios of missingness during the imputation procedure. The imputation will be done separately for each intervention arm. In addition, a per-protocol analysis (defined as ≥ 75% attendance rate as well as no exacerbations during the last 2 weeks prior to follow-up assessment), and a complete case analysis (including participants with complete outcome measurements independent of attendance rate) will be reported.

### Monitoring and safety

Participants will be monitored by instructors and assessors during training sessions and outcome assessments to identify symptoms or signals that would require interruption of the session or exclusion from the trial. Blood oxygen saturation and blood pressure measurements will be taken if deemed necessary. The trial centre teams have had on-site visits prior to the start of the intervention to ensure standardization across centres. Research progress will be monitored closely, and adjustments to individual tasks will be made if necessary, using monthly group meetings across participating centres.

Reasons for immediately stopping an outcome assessment or training session, and examples of possible adverse events, include chest pain, intolerable dyspnoea, leg cramps, staggering, dizziness, diaphoresis, and pale or ashen appearance. When monitored via ECG, blood pressure, and saturation during cycling tests, additional indications to terminate exercise are defined [133].

An external data monitoring and safety committee (DMC) will consist of an independent group of medical doctors and physiotherapists that will monitor patient safety and treatment efficacy. All adverse events will be reported to the DMC every third month of the project, which could result in the discontinuation of the trial for the individual participant. The DMC will be responsible for decisions related to stopping the trial if any SAE happens during the trial period. There are no interim analyses planned other than the sample size re-estimation in phase 2. There is no other pre-planned auditing of the trial. The funders might do project reviews and financial audits, independent from the sponsor.

#### Protocol amendments

Any modifications to the protocol which may impact the conduct of the trial, potential benefit of the patient or may affect patient safety, including changes of study objectives, trial design, participant population, sample sizes, trial procedures, statistical analyses, or significant administrative aspects will require a formal amendment to the protocol, or if adequate, be included in the SAP. Such amendments will be agreed upon by the research group with the final decision by the PI and, if needed, approved by the Regional/Local Ethics Committees. Administrative changes of the protocol (e.g., minor corrections and/or clarifications) that have no effect on how the trial is conducted will be agreed upon by the research group with the final decision by the PI and documented and presented upon publication.

#### Ancillary and post-trial care

Participants that are enrolled into the trial are covered by a local insurance that will cover potential additional healthcare needs that arise because of trial participation, such as adverse events during the intervention.

### Access to data

Access to data is based on the established collaboration agreements including material and data transfer agreements.

### Dissemination plans

The results from this trial will be disseminated regardless of the magnitude or direction of effect. Results from this trial will be submitted for publication in peer-reviewed journals and presented at national and international conferences (e.g., European Respiratory Society Congress). We will also disseminate the results via newsletter articles, social media, and talks to clinicians, patient organizations, individuals with COPD and older healthy adults. Trial participants who are interested in receiving a summary of their own results will receive them via mail.

Actors who have contributed substantially in study design, data collection, data analysis and interpretation, manuscript preparation and critical revising, and in final manuscript version approval will be affiliated as authors according to international recommendations [134].

## Discussion

We have designed a comprehensive multicentre randomized controlled trial to investigate the effects of supramaximal HIIT compared to MICT in people with COPD and healthy controls. It is the first comprehensive trial investigating supramaximal HIIT in the COPD population. COPD-HIIT is also the first trial to, in a large sample of people with COPD, investigate global cognitive function using an extensive test battery [27], and brain health using both MRI and PET/CT scans.

The COPD-HIIT project has the potential to induce a shift in the therapeutic approach to extrapulmonary manifestations in COPD. Currently, no therapeutic modality available for COPD can effectively target multiple manifestations of the disease [9]. The advantage and novelty of the supramaximal HIIT approach entails the enabling of participants to reach supramaximal exercise intensities in a safe and feasible way. In addition to exercising at higher intensity, reducing the ventilatory burden with supramaximal HIIT will potentially allow people with COPD to experience multiple extrapulmonary benefits. This is partly via effective activation of the AMPK-PGC-1α-BDNF pathway, beneficial for mitochondrial and brain health. Also, the supramaximal HIIT modality is promising for increased adherence given that it might reduce dyspnoea, is shorter in duration compared to MICT, and in our preliminary data, was preferred by most participants [17].

A strength in the COPD-HIIT project is the international and interdisciplinary research collaboration with state-of-the-art infrastructures. Additionally, our trial’s inclusion of objectively measured physical activity matched healthy controls distinguishes itself from prior research on exercise and extrapulmonary manifestations in COPD. This feature will contribute valuable insights to the ongoing debate regarding the impact of COPD on exercise response and adaptation [4, 135] . Another strength of the trial is the carefully planned intervention, guided by the fundamental training principles and the CERT checklist [42].

As all randomized controlled trials, this trial has some challenges and limitations. While different exercise intensities will be investigated, we will not be able to determine the optimal dose of the exercise regimens, but only draw conclusions on the prescribed doses. Maintaining a high attendance over the course of 2 years is a challenge. Trial withdrawals and missing data, as well as random differences at baseline are factors than can interfere with the interpretation of the intervention’s effects. However, data imputation, randomization, and adjustment for covariates will to some extent mitigate these challenges.

## Trial status

This manuscript reports protocol version 1.1 finalized at 11 April 2024. Ethical approval has been obtained for all procedures in Umeå, Sweden, and in Hasselt, Belgium. The trial started enrolling participants on the 9th of November 2023 in Umeå, Sweden and on the 16th of September 2024 in Hasselt, Belgium. At the time of manuscript submission (3 January 2024), 14 participants had been enrolled. At manuscript acceptance, 17th September 2024, 51 participants have been enrolled. Data collection for phase 1 is expected to take place over 2–3 years, while phase 2 is expected to take place over 5 years.

## Supplementary Information

Additional file 1: SPIRIT checklist.

Additional file 2: TIDieR Checklist.

Additional file 3: CERT Checklist.

Additional file 4: Supplementary information.

Additional file 5: Figure S1-S3.

Additional file 6: SPIRIT Item 2b WHO Trial Data Set.

Additional file 7: Model consent form COPD English.

## Data Availability

We aim to share all the de-identified individual participant data that underlie results in a publication in an open data repository, if this will be possible given regulations and agreements at the time. Data will be shared 6-months after publication.

## Abbreviations

5-STS: Five times sit-to-stand test
AE: Adverse event
AMPK: Adenosine monophosphate-activated protein kinase
ATS: American Thoracic Society
BCST: Borg cycle strength test
BDNF: Brain-derived neurotrophic factor
BMI: Body mass index
CAT: COPD Assessment Test
CERT: Consensus on exercise reporting template
CR10: Category ratio 10
CRF: Case report form
CRQ: Chronic respiratory disease questionnaire
COPD: Chronic obstructive pulmonary disease
CONSORT: Consolidated standard reporting of trials
CVD: Cardiovascular disease
CPET: Cardiopulmonary exercise test
CS: Citrate synthase
DTI: Diffusion tensor imaging
DMC: Data monitoring committee
ECG: Electrocardiogram
EDTA: Ethylenediaminetetraacetic
ELISA: Enzyme-linked immunosorbent assay
ERS: European Respiratory Society
EQ: EuroQol
F: Force
FLAIR: Fluid-attenuated inversion recovery
FNDC5: Fibronectin type III domain-containing protein 5
GOLD: Global initiative for chronic lung disease
HADS: Hospital Anxiety and Depression Scale
HADH: 3-Hydroxyacyl coenzyme A dehydrogenase
HbA1C: Glycated haemoglobin
HIIT: High-intensity interval training
HDL: High-density lipoprotein
HRQoL: Health-related quality of life
HRV: Heart rate variability
hs-CRP: High-sensitivity C-reactive protein
IGF-1: Insulin-like growth factor 1
IL: Interleukin
KYN: Kynurenine
KYNA: Kynurenine acid
KAT1-4: Kynurenine aminotransferase
LDH: Lactate dehydrogenase
LDL: Low-density lipoprotein
MAP: Maximal aerobic power
MCID: Minimal clinically important difference
MDC: Minimal detectable change
MICT: Moderate-intensity continuous training
mMRC: Modified Medical Research Council Dyspnea scale
MRI: Magnetic resonance imaging
MoCA: Montreal Cognitive Assessment
MPO_6_: Maximum mean power output for 6 s
NRF1: Nuclear respiratory factor 1
NRF2: Nuclear respiratory factor 2
P: Power
PET/CT: Positron emission tomography/computed tomography
PGC-1α: Peroxisome proliferator-activated receptor gamma coactivator 1-alpha
PGC-1β: Peroxisome proliferator-activated receptor gamma coactivator 1-beta
PFK: Phosphofructokinase
PPAR-α: Peroxisome proliferator-activated receptor-alpha
qPCR: Quantitative polymerase chain reaction
REDCap: Research electronic data capture
RHR: Resting heart rate
RMSSD: Root of the mean square of successive R–R interval differences
RPE: Rating of perceived exertion
SAE: Severe adverse event
SAP: Statistical analysis plan
SCPT: Stair climb power test
SDH: Succinate dehydrogenase
SDNN: Standard deviation of N–N intervals
SPIRIT: Standard Protocol Items: Recommendations for Interventional Trials
SST: Serum separator tube
TFAM: Mitochondrial transcription factor A
TIDieR: Template for intervention description and replication
TNF-α: Tumour necrosis factor-alpha
V: Velocity
VEGF: Vascular endothelial growth factor
VO_2_peak: Peak oxygen consumption
