# Correction: Optimising AVATAR therapy for people who hear distressing voices: study protocol for the AVATAR2 multi-centre randomised controlled trial

**DOI:** 10.1186/s13063-024-08550-7

**Published:** 2024-12-17

**Authors:** Philippa Garety, Clementine J. Edwards, Thomas Ward, Richard Emsley, Mark Huckvale, Paul McCrone, Mar Rus-Calafell, Miriam Fornells-Ambrojo, Andrew Gumley, Gillian Haddock, Sandra Bucci, Hamish McLeod, Amy Hardy, Emmanuelle Peters, Inez Myin-Germeys, Thomas Craig

**Affiliations:** 1 Institute of Psychiatry, Psychology & Neuroscience, King’s College London, London, UK; 2 South London & Maudsley NHS Foundation Trust, London, UK; 3 University College London, London, UK; 4 University of Greenwich, London, UK; 5 Mental Health Research and Treatment Center, Faculty of Psychology, Ruhr-Universität Bochum, Bochum, Germany; 6 South London & Maudsley NHS Foundation Trust, London, UK; 7 University of Glasgow, Glasgow, UK; 8 NHS Greater Glasgow & Clyde, Glasgow, UK; 9 University of Manchester and the Manchester Academic Health Sciences Centre, Manchester, UK; 10 Greater Manchester Mental Health NHS Foundation Trust and the Manchester Academic Health Sciences Centre, Manchester, UK; 11 KU Leuven, Leuven, Belgium

**Correction** **:** **Trials 22, 366 (2021)**

**https://doi.org/10.1186/s13063-021-05301-w**

Following the publication of the original article [1], we were notified that the reference numbers provided in Table 1 were incorrect.

Originally published Table 1:

**Table:**
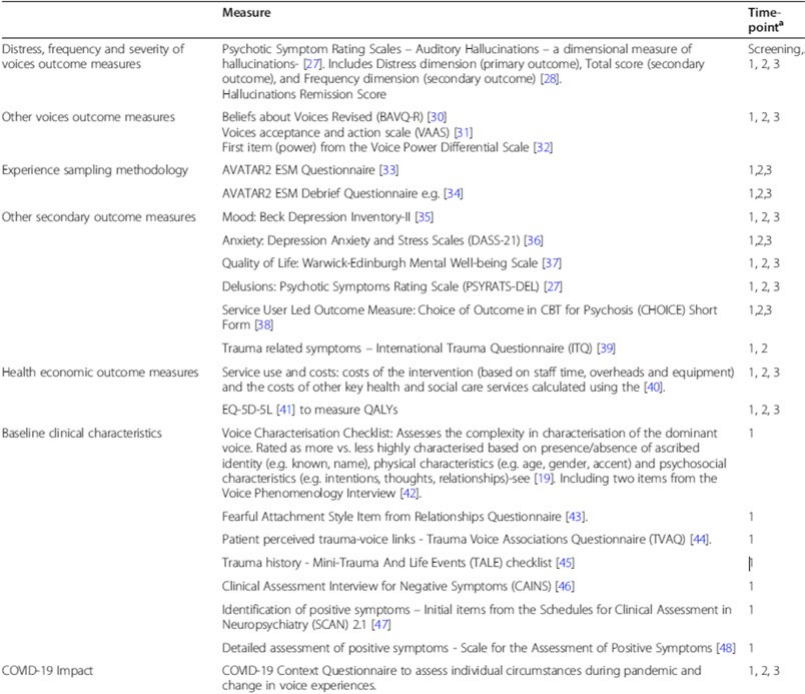

Corrected Table 1:

**Table:** 

	Measure	Time-point*
Distress, Frequency and Severity of Voices Outcome Measures	Psychotic Symptom Rating Scales – Auditory Hallucinations – a dimensional measure of hallucinations [29]. Includes Distress dimension (primary outcome), Total score (secondary outcome), and Frequency dimension (secondary outcome) [30]. Hallucinations Remission Score	Screening, 1, 2, 3
Other Voices Outcome Measures	Beliefs about Voices Revised (BAVQ-R) [32]. Voices acceptance and action scale (VAAS) [33]. First item (power) from the Voice Power Differential Scale [34].	1, 2, 3
Experience Sampling Methodology	AVATAR2 ESM Questionnaire [35].	1,2,3
	AVATAR2 ESM Debrief Questionnaire [36].	1,2,3
Other Secondary Outcome Measures	Mood: Beck Depression Inventory-II [37].	1, 2, 3
	Anxiety: Depression Anxiety and Stress Scales (DASS-21) [38].	1,2,3
	Quality of Life: Warwick-Edinburgh Mental Well-being Scale [39].	1, 2, 3
	Delusions: Psychotic Symptoms Rating Scale (PSYRATS-DEL) [29].	1, 2, 3
	Service User Led Outcome Measure: Choice of Outcome in CBT for Psychosis (CHOICE) Short Form [40].	1,2,3
	Trauma related symptoms – International Trauma Questionnaire (ITQ) [41].	1, 2
Health Economic Outcome Measures	Service use and costs: costs of the intervention (based on staff time, overheads and equipment) and the costs of other key health and social care services calculated using the [42].	1, 2, 3
	EQ-5D-5L [43] to measure QALYs	1, 2, 3
Baseline Clinical Characteristics	Voice Characterisation Checklist: Assesses the complexity in characterisation of the dominant voice. Rated as more vs. less highly characterised based on presence/absence of ascribed identity (e.g. known, name), physical characteristics (e.g. age, gender, accent) and psychosocial characteristics (e.g. intentions, thoughts, relationships)-see [21]. Including two items from the Voice Phenomenology Interview [44].	1
	Fearful Attachment Style Item from Relationships Questionnaire [45].	1
	Patient perceived trauma-voice links - Trauma Voice Associations Questionnaire (TVAQ)	1
	Trauma history - Mini-Trauma And Life Events (TALE) checklist [46].	1
	Clinical Assessment Interview for Negative Symptoms (CAINS) [47].	1
	Identification of positive symptoms – Initial items from the Schedules for Clinical Assessment in Neuropsychiatry (SCAN) 2.1 [48].	1
	Detailed assessment of positive symptoms - Scale for the Assessment of Positive Symptoms [49].	1
Covid-19 Impact	Covid-19 Context Questionnaire to assess individual circumstances during pandemic and change in voice experiences.	1, 2, 3

The original article has been corrected.
