# Supplementary material for: Effects and mechanisms of supramaximal high-intensity interval training on extrapulmonary manifestations in people with and without chronic obstructive pulmonary disease (COPD-HIIT): study protocol for a multi-centre, randomized controlled trial
Source: Trials. 2024 Oct 8;25:664. doi: 10.1186/s13063-024-08481-3 (PMC11460198; doi:10.1186/s13063-024-08481-3)
Supplement: Supplementary file 1 — Additional file 1: SPIRIT checklist. [file 13063_2024_8481_MOESM1_ESM.pdf]

SPIRIT Checklist for *Trials*

Complete this checklist by entering the page and line numbers where each of the items listed below can be found in your manuscript.

Your manuscript may not currently address all the items on the checklist. Please modify your text to include the missing information. If you are certain that an item does not apply, please state "n/a" and provide a short explanation. **Leaving an item blank or stating "n/a" without an explanation will lead to your manuscript being returned before review.**

Upload your completed checklist as an additional file when you submit to *Trials*. You must reference this additional file in the main text of your protocol submission. The completed SPIRIT figure must be included within the main body of the protocol text and can be downloaded here: http://www.spirit-statement.org/schedule-of-enrolment-interventions-and-assessments/

In your methods section, please state that you used the SPIRIT reporting guidelines, and cite them as:

Chan A-W, Tetzlaff JM, Gøtzsche PC, Altman DG, Mann H, Berlin J, Dickersin K, Hróbjartsson A, Schulz KF, Parulekar WR, Krleža-Jerić K, Laupacis A, Moher D. SPIRIT 2013 Explanation and Elaboration: Guidance for protocols of clinical trials. BMJ. 2013;346:e7586

		Reporting Item	Page and Line Number	Reason if not applicable
**Administrative information**				
Title	#1	Descriptive title identifying the study design, population, interventions, and, if applicable, trial acronym	Page 1, Line 3-5	
Trial registration	#2a	Trial identifier and registry name. If not yet registered, name of intended registry	Page 3, Line 48	
Trial registration: data set	#2b	All items from the World Health Organization Trial Registration Data Set	Additional file 6	
Protocol version	#3	Date and version identifier	Page 3, Line 50	

Funding	#4	Sources and types of financial, material, and other support	Page 3, Line 52-57<br><br>Page 59, Line 1372-1378	
Roles and responsibilities: contributorship	#5a	Names, affiliations, and roles of protocol contributors	Page 1, Line 7-9<br><br>Page 1-2, Line 10-39<br><br>Page 57-59, Line 1323-1370	
Roles and responsibilities: sponsor contact information	#5b	Name and contact information for the trial sponsor	Page 2, Line 41-43	
Roles and responsibilities: sponsor and funder	#5c	Role of study sponsor and funders, if any, in study design; collection, management, analysis, and interpretation of data; writing of the report; and the decision to submit the report for publication, including whether they will have ultimate authority over any of these activities	Page 3, Line 45-46<br><br>Page 3, Line 55-57<br><br>Page 59, Line 1377-1378	
Roles and responsibilities: committees	#5d	Composition, roles, and responsibilities of the coordinating centre, steering committee, endpoint adjudication committee, data management team, and other individuals or groups overseeing the trial, if applicable (see Item 21a for data monitoring committee)	Page 50, Line 1164-1168	
**Introduction**			Page 6-11, Line 107-242	
Background and rationale	#6a	Description of research question and justification for undertaking the trial, including summary of	Page 6-9, Line 108-195	

		relevant studies (published and unpublished) examining benefits and harms for each intervention		
Background and rationale: choice of comparators	#6b	Explanation for choice of comparators	Page 6, Line 125-131<br>Page 7, Line 142-145	
Objectives	#7	Specific objectives or hypotheses	Page 9-10, Line 196-222<br>Additional file, Figure 1	
Trial design	#8	Description of trial design including type of trial (eg, parallel group, crossover, factorial, single group), allocation ratio, and framework (eg, superiority, equivalence, non-inferiority, exploratory)	Page 10-11, Line 223-242<br>Additional file, Figure 1 and 2	
**Methods: Participants, interventions, and outcomes**				
Study setting	#9	Description of study settings (eg, community clinic, academic hospital) and list of countries where data will be collected. Reference to where list of study sites can be obtained	Page 11-12, Line 244-249	
Eligibility criteria	#10	Inclusion and exclusion criteria for participants. If applicable, eligibility criteria for study centres and individuals who will perform the interventions (eg, surgeons, psychotherapists)	Page 12-14, Line 275-316	
Interventions: description	#11a	Interventions for each group with sufficient detail to allow replication, including how and when they will be administered	Page 14-19, Line 319-429<br>Page 20, Table 1	

			Page 24, Table 2<br><br>Additional file, Figure 2<br><br>Additional file, Table 3<br><br>Additional file, Table 5<br><br>Page 25, Table 4	
Interventions: modifications	#11b	Criteria for discontinuing or modifying allocated interventions for a given trial participant (eg, drug dose change in response to harms, participant request, or improving / worsening disease)	Page 19, Line 430-435<br><br>Page 21, Line 474-478<br><br>Page 21, Line 484-485<br><br>Page 22, Line 501-502	
Interventions: adherance	#11c	Strategies to improve adherence to intervention protocols, and any procedures for monitoring adherence (eg, drug tablet return; laboratory tests)	Page 21-22, Line 462-502	
Interventions: concomitant care	#11d	Relevant concomitant care and interventions that are permitted or prohibited during the trial	Page 22-23, Line 504-516	
Outcomes	#12	Primary, secondary, and other outcomes, including the specific measurement variable (eg, systolic blood pressure), analysis metric (eg, change from baseline, final value, time to event), method of aggregation (eg, median, proportion), and time point for each outcome. Explanation of the clinical relevance of chosen efficacy and harm outcomes is strongly recommended	Page 28-44, Line 610-1014<br><br>Additional file, Table 6	

Participant timeline	#13	Time schedule of enrolment, interventions (including any run-ins and washouts), assessments, and visits for participants. A schematic diagram is highly recommended (see Figure)	Additional file, Figure 2<br><br>Additional file, Table 5	
Sample size	#14	Estimated number of participants needed to achieve study objectives and how it was determined, including clinical and statistical assumptions supporting any sample size calculations	Page 44-46, Line 1016-1060	
Recruitment	#15	Strategies for achieving adequate participant enrolment to reach target sample size	Page 12, Line 259-271	
**Methods: Assignment of interventions (for controlled trials)**				
Allocation: sequence generation	#16a	Method of generating the allocation sequence (eg, computer-generated random numbers), and list of any factors for stratification. To reduce predictability of a random sequence, details of any planned restriction (eg, blocking) should be provided in a separate document that is unavailable to those who enrol participants or assign interventions	Page 46, Line 1062-1065	
Allocation concealment mechanism	#16b	Mechanism of implementing the allocation sequence (eg, central telephone; sequentially numbered, opaque, sealed envelopes), describing any steps to conceal the sequence until interventions are assigned	Page 46, Line 1066-1069	

Allocation: implementation	#16c	Who will generate the allocation sequence, who will enrol participants, and who will assign participants to interventions	Page 46, Line 1069-1071	
Blinding (masking)	#17a	Who will be blinded after assignment to interventions (eg, trial participants, care providers, outcome assessors, data analysts), and how	Page 46-47, Line 1072-1077	
Blinding (masking): emergency unblinding	#17b	If blinded, circumstances under which unblinding is permissible, and procedure for revealing a participant's allocated intervention during the trial	NA	As trial participants and intervention providers are not blinded to the allocated intervention, there are no circumstances under which unblinding is permissible
**Methods: Data collection, management, and analysis**				
Data collection plan	#18a	Plans for assessment and collection of outcome, baseline, and other trial data, including any related processes to promote data quality (eg, duplicate measurements, training of assessors) and a description of study instruments (eg, questionnaires, laboratory tests) along with their reliability and validity, if known. Reference to where data collection forms can be found, if not in the protocol	Page 26-44, Line 551-1014<br><br>Additional file, Table 6<br><br>Page 47, Line 1080-1090<br><br>Page 60, Line 1394-1396	
Data collection plan: retention	#18b	Plans to promote participant retention and complete follow-up, including list of any outcome data to be collected for participants who discontinue or deviate from intervention protocols	Page 19, Line 432-437<br><br>Page 21-22, Line 460-502	

Data management	#19	Plans for data entry, coding, security, and storage, including any related processes to promote data quality (eg, double data entry; range checks for data values). Reference to where details of data management procedures can be found, if not in the protocol	Page 47, Line 1080-1099	
Statistics: outcomes	#20a	Statistical methods for analysing primary and secondary outcomes. Reference to where other details of the statistical analysis plan can be found, if not in the protocol	Page 48-49, Line 1102-1138	
Statistics: additional analyses	#20b	Methods for any additional analyses (eg, subgroup and adjusted analyses)	Page 49, Line 1140-1142	
Statistics: analysis population and missing data	#20c	Definition of analysis population relating to protocol non-adherence (eg, as randomised analysis), and any statistical methods to handle missing data (eg, multiple imputation)	Page 49-50, Line 1144-1152	
**Methods: Monitoring**				
Data monitoring: formal committee	#21a	Composition of data monitoring committee (DMC); summary of its role and reporting structure; statement of whether it is independent from the sponsor and competing interests; and reference to where further details about its charter can be found, if not in the protocol. Alternatively, an explanation of why a DMC is not needed	Page 50, Line 1164-1168	

Data monitoring: interim analysis	#21b	Description of any interim analyses and stopping guidelines, including who will have access to these interim results and make the final decision to terminate the trial	Page 50, Line 1167-1168	
Harms	#22	Plans for collecting, assessing, reporting, and managing solicited and spontaneously reported adverse events and other unintended effects of trial interventions or trial conduct	Page 43, Line 992-996<br><br>Additional file, Table 5<br><br>Additional file 4, supplementary information, Page 3-8	
Auditing	#23	Frequency and procedures for auditing trial conduct, if any, and whether the process will be independent from investigators and the sponsor	Page 50, 1170-1171.	
**Ethics and dissemination**				
Research ethics approval	#24	Plans for seeking research ethics committee / institutional review board (REC / IRB) approval	Page 59, Line 1387-1392<br><br>See attached ethical approval files	
Protocol amendments	#25	Plans for communicating important protocol modifications (eg, changes to eligibility criteria, outcomes, analyses) to relevant parties (eg, investigators, REC / IRBs, trial participants, trial registries, journals, regulators)	Page 50-51, Line 1169-1178	
Consent or assent	#26a	Who will obtain informed consent or assent from potential trial participants or authorised surrogates, and how (see Item 32)	Page 12, Line 272-273<br><br>Page 59, Line 1387 - 1388	

Consent or assent: ancillary studies	#26b	Additional consent provisions for collection and use of participant data and biological specimens in ancillary studies, if applicable	Page 59, Line 1388-1390	
Confidentiality	#27	How personal information about potential and enrolled participants will be collected, shared, and maintained in order to protect confidentiality before, during, and after the trial	Page 47, Line 1091-1099	
Declaration of interests	#28	Financial and other competing interests for principal investigators for the overall trial and each study site	Page 60, Line 1398-1399	
Data access	#29	Statement of who will have access to the final trial dataset, and disclosure of contractual agreements that limit such access for investigators	Page 51, Line 1183-1185<br>Page 59, Line 1379-1382	
Ancillary and post trial care	#30	Provisions, if any, for ancillary and post-trial care, and for compensation to those who suffer harm from trial participation	Page 51, Line 1179-1182	
Dissemination policy: trial results	#31a	Plans for investigators and sponsor to communicate trial results to participants, healthcare professionals, the public, and other relevant groups (eg, via publication, reporting in results databases, or other data sharing arrangements), including any publication restrictions	Page 51, Line 1186-1192<br>Page 59, Line 1379-1382<br>Page 60, Line 1394-1396	
Dissemination policy: authorship	#31b	Authorship eligibility guidelines and any intended use of professional writers	Page 51, Line 1193-1195<br>Page 57, Line 1323-1324	

Dissemination policy: reproducible research	#31c	Plans, if any, for granting public access to the full protocol, participant-level dataset, and statistical code	Page 59, Line 1379-1382	
**Appendices**				
Informed consent materials	#32	Model consent form and other related documentation given to participants and authorised surrogates	NA	Informed consent forms (in local language) in local language can be sent / uploaded upon request
Biological specimens	#33	Plans for collection, laboratory evaluation, and storage of biological specimens for genetic or molecular analysis in the current trial and for future use in ancillary studies, if applicable	Page 35-37, Line 790-834<br><br>Page 39-41, Line 885-928	

It is strongly recommended that this checklist be read in conjunction with the SPIRIT 2013 Explanation & Elaboration for important clarification on the items. Amendments to the protocol should be tracked and dated. The SPIRIT checklist is copyrighted by the SPIRIT Group under the Creative Commons "Attribution-NonCommercial-NoDerivs 3.0 Unported" license. This checklist can be completed online using https://www.goodreports.org/, a tool made by the EQUATOR Network in collaboration with Penelope.ai
