# Supplementary material for: Effects and mechanisms of supramaximal high-intensity interval training on extrapulmonary manifestations in people with and without chronic obstructive pulmonary disease (COPD-HIIT): study protocol for a multi-centre, randomized controlled trial
Source: Trials. 2024 Oct 8;25:664. doi: 10.1186/s13063-024-08481-3 (PMC11460198; doi:10.1186/s13063-024-08481-3)
Supplement: Supplementary file 3 — Additional file 3: CERT Checklist. [file 13063_2024_8481_MOESM3_ESM.pdf]

CERT ✓ Consensus on Exercise Reporting Template

A Checklist for what to include when reporting exercise programs

Section/Topic	Item #	Checklist item	Location ** Primary paper (page, table, appendix)	† Other (paper or protocol, website (URL)
**WHAT: materials**	1	Detailed description of the type of exercise equipment (e.g. weights, exercise equipment such as machines, treadmill, bicycle ergometer etc)	14, 17, Table 3-4	
**WHO: provider**	2	Detailed description of the qualifications, teaching/supervising expertise, and/or training undertaken by the exercise instructor	15, Table 3	
**HOW: delivery**	3	Describe whether exercises are performed individually or in a group	14, 17-18 ,Table 3-4	
	4	Describe whether exercises are supervised or unsupervised and how they are delivered	14-15, 17-18 Table 3-4,	
	5	Detailed description of how adherence to exercise is measured and reported	21-22, 42-43, Table 3-4	
	6	Detailed description of motivation strategies	21-22	
	7a	Detailed description of the decision rule(s) for determining exercise progression	15-17, Table 1,2,4	
	7b	Detailed description of how the exercise program was progressed	15-17, Table 1,2,4	
	8	Detailed description of each exercise to enable replication (e.g. photographs, illustrations , video etc)	15-18, Table 1-4 ,Additional file 5	
	9	Detailed description of any home program component (e.g. other exercises, stretching etc)	18, Table 4, Additional file 5	
	10	Describe whether there are any non-exercise components (e.g. education, cognitive behavioural therapy, massage etc)	N/A	
	11	Describe the type and number of adverse events that occurred during exercise	N/A	

**WHERE: location**	12	Describe the setting in which the exercises are performed	14-15,17-18 Table 3-4
**WHEN, HOW MUCH: dosage**	13	Detailed description of the exercise intervention including, but not limited to, number of exercise repetitions/sets/sessions, session duration, intervention/program duration etc	14-18, Table 1-4
**TAILORING: what, how**	14a	Describe whether the exercises are generic (one size fits all) or tailored whether tailored to the individual	14-17, Table 1-4, Additional file 5
	14b	Detailed description of how exercises are tailored to the individual	15-17, Table 1-4, Additional file 5
	15	Describe the decision rule for determining the starting level at which people commence an exercise program (such as beginner, intermediate, advanced etc)	15-17, Table 1-4, Additional file 5
**HOW WELL: planned, actual**	16a	Describe how adherence or fidelity to the exercise intervention is assessed/measured	42-43
	16b	Describe the extent to which the intervention was delivered as planned	N/A

***It is recommended that this checklist is used in conjunction with the Explanation and Elaboration Statement which is a guide each item in the CERT Checklist**

The CERT Checklist is designed for reporting details of an exercise intervention. The CERT Checklist should be used in conjunction with a reporting checklist appropriate for the study type e.g. the CONSORT Statement (www.consort-statement.org) for randomised controlled trials, the SPIRIT Statement (www.spirit-statement.org) for a clinical trial protocol. For further guidance regarding reporting guidelines please consult the EQUATOR network (www.equator-network.org)

** Authors – please use N/A if an item is not applicable Reviewers – please use ”?” if information is not provided or not/insufficiently reported

† If the information is not provided in the primary paper that is under consideration, please provide details of where this information is available e.g. in a published protocol, published papers (provide citation details) or on a website (provide the URL).
