# Supplementary material for: Effects and mechanisms of supramaximal high-intensity interval training on extrapulmonary manifestations in people with and without chronic obstructive pulmonary disease (COPD-HIIT): study protocol for a multi-centre, randomized controlled trial
Source: Trials. 2024 Oct 8;25:664. doi: 10.1186/s13063-024-08481-3 (PMC11460198; doi:10.1186/s13063-024-08481-3)
Supplement: Supplementary file 4 — Additional file 4: Supplementary information. [file 13063_2024_8481_MOESM4_ESM.docx]

# **COPD-HIIT Trial online supplement for study protocol**

## RESISTANCE TRAINING EXERCISES

See PDF figures S1-S3 in additional file 5.

## CYCLE TEST EQUIPMENT

Table S1: Overview of cycle test equipment and manufacturers at both study sites.

| Study site | UMEÅ | | HASSELT |
| --- | --- | --- | --- |
| Cycle test | CPET | BCST/CWRT | CPET/BCST/CWRT |
| Ergometer | **Lode Corival cpet**  *Lode, Groningen, The Netherlands* | **Lode Corival cpet**  *Lode, Groningen, The Netherlands* | **Lode Corival cpet**  *Lode, Groningen, The Netherlands* |
| Metabolic cart | **Vyntus CPX**  *Vyaire Medical, Mettawa, USA* | **Vyntus CPX**  *Vyaire Medical, Mettawa, USA* | **Vyntus CPX**  *Vyaire Medical, Mettawa, USA* |
| Heart rate | **Cardiolex EC Sense (12-lead ECG)**  *Cardiolex Medical, Solna, Sweden* | **Polar H9 or H10 (chest strap)**  *Polar Electro, Kempele, Finland* | **Vyntus ECG** - CPET  *Vyaire Medical, Mettawa, USA*  **Polar H9 or H10 (chest strap) -** **BCST/CWRT**  *Polar Electro, Kempele, Finland* |
| Oxygen saturation | **Vyntus SpO_2_ sensor (forehead)**  *Vyaire Medical, Mettawa, USA* | **Vyntus SpO_2_ sensor (earlobe)**  *Vyaire Medical, Mettawa, USA* | **Vyntus SpO_2_ sensor (earlobe)**  *Vyaire Medical, Mettawa, USA* |
| Blood pressure | ***Sphygmomanometer***  *AB Henry Eriksson, Bandhagen, Sweden* | **Tango M2**  *SunTech Medical, Morrisville, USA* | **Lode**  *Lode, Groningen, The Netherlands* |
| ECG | **Cardiolex EC Sense**  *Cardiolex Medical, Solna, Sweden* | **N/A** | **Vyntus ECG**  *Vyaire Medical, Mettawa, USA* |

Note: The SentrySuite software will be used during tests with the Vyntus CPX. ECG = electrocardiogram.

## LUNG FUNCTION EQUIPMENT

Table S2: Lung function testing equipment and manufacturers at both study sites.

| Study site | UMEÅ | HASSELT |
| --- | --- | --- |
| Spirometry | **Vyntus One**  *Vyaire Medical, Mettawa, USA* | ***Masterscreen PFT / Vyntus One****  *Vyaire Medical, Mettawa, USA* |
| Body plethysmography | **Vyntus Body**  *Vyaire Medical, Mettawa, USA* | **Masterscreen Bodybox / Vyntus Body*****  *Vyaire Medical, Mettawa, USA* |
| Diffusion capacity | **Vyntus One**  *Vyaire Medical, Mettawa, USA* | ***Masterscreen PFT / Vyntus One****  *Vyaire Medical, Mettawa, USA* |

Note: ***** = to be decided

## SATISFACTION SURVEY

**Satisfaction survey for those who performed supramaximal HIIT:**

*During these 12 weeks, you have completed exercise training in the form of* ***high-intensity interval cycling****, where after a 5-minute warm-up, you have completed short intervals at a high intensity followed by rest breaks between the intervals. You have then finished the workouts with a 5-minute cool-down.*

*We would like you to answer a few short questions regarding the training.*

**Table S3: Satisfaction survey – supramaximal HIIT in COPD**

| **Questions** | **Strongly agree** | **Agree** | **Neutral** | **Disagree** | **Strongly Disagree** |
| --- | --- | --- | --- | --- | --- |
| *I understand why I was asked to perform high-intensity interval cycling* |  |  |  |  |  |
| *I found high-intensity interval cycling easy to learn* |  |  |  |  |  |
| *I found high-intensity interval cycling helped to exercise/be physically active* |  |  |  |  |  |
| *I enjoyed high-intensity interval cycling* |  |  |  |  |  |
| *I would recommend high-intensity interval cycling to other people with COPD* |  |  |  |  |  |
| *I would like to continue with high-intensity interval cycling* |  |  |  |  |  |

**Satisfaction survey for those who performed moderate-intensity continuous cycling:**

*During these 12 weeks, you have completed cardio training in the form of* ***moderate-intensity continuous cycling****, where after a 5-minute warm-up, you have completed a continuous workout without breaks. You have then finished the workouts with a 5-minute cool-down.*

*We would like you to answer a few short questions regarding the training.*

**Table S4: Satisfaction survey – MICT in COPD**

| **Questions** | **Strongly agree** | **Agree** | **Neutral** | **Disagree** | **Strongly Disagree** |
| --- | --- | --- | --- | --- | --- |
| *I understand why I was asked to perform moderate-intensity continuous cycling* |  |  |  |  |  |
| *I found moderate-intensity continuous cycling easy to learn* |  |  |  |  |  |
| *I found moderate-intensity continuous cycling helped to exercise/be physically active* |  |  |  |  |  |
| *I enjoyed moderate-intensity continuous cycling* |  |  |  |  |  |
| *I would recommend moderate-intensity continuous cycling to other people with COPD* |  |  |  |  |  |
| *I would like to continue with moderate-intensity continuous cycling* |  |  |  |  |  |

## STANDARDIZED PHONE CALL: ATTENDANCE & ADHERENCE (INTERVENTION GROUPS)

**Flow for phone call (every 14^th^ day)**

- How has it been for you since our last conversation (both positive/negative)?
  - How did the training sessions generally feel during the last two weeks?
  - Other important remarks since the last call?
- How many resistance training sessions were performed in total in the last 14 days?

____ out of 4 = ____ % attendance

- Use the table below and ask the participant about their notes from the last performed session. Note the number of sets the participant performed per exercise, performed repetitions in the last set, dyspnea ratings and ratings of perceived heaviness in the last set in every exercise *(an example is given in one of the tables).*

| **Date:** 2023-01-02 | | | | |
| --- | --- | --- | --- | --- |
| **Exercises** | **Sets** | **Reps** | **Dyspnea** | **Heaviness** |
| Sit to stand level 1 | 2 | 12 | 4 | 6 |
| Calf raises | 2 | 12 | 4 | 6 |
| Chest press | 2 | 10 | 5 | 5 |
| Shoulder flexion | 2 | 10 | 5 | 5 |

- How many endurance sessions were performed in total the last 14 days?

___ out of 4 =____ % attendance

| **Date** | **Intervals** | **Dyspnea** | **General perceived exertion (RPE)** |
| --- | --- | --- | --- |
| *HIIT walk 01/01/2023* | *10* | *5* | *14* |
|  |  |  |  |
|  |  |  |  |
|  |  |  |  |

- Use the table below and ask the participant about their notes from the last performed session. *(an example is given in the table).*
- Based on the participant information you will provide the training scheme for the next two weeks
  - Endurance training: intervals and time
  - Resistance training: selection of exercise level/load, sets, reps
  - Other important remarks from the call?

## TRAINING DIARY IN PHASE 2 (INTERVENTION GROUPS)

### INSTRUCTIONS

***Resistance training:***

Start with choosing the **four** exercises that you want to perform. Below you can see instructions on how to perform the exercises depending on the goal (you will receive a new goal every month).

| **Goal** | **Sets** | **Reps** | **Tempo** | **Rest between sets** | **Rest between exercises** |
| --- | --- | --- | --- | --- | --- |
| Strength | 2 | 8-12 | Let your breathing guide your tempo.  **For example**, for the sit-to-stand exercise: breathe in while standing up and breathe out while sitting down. | 1 min | 4 min |
| Endurance | 2 | 15-25 |  | 1 min | 4 min |
| Power | 2 | 8-12 | As fast as you can/are comfortable with in the first phase of the movement, slow in the second phase.  **For example**, for the sit-to-stand exercise: stand up as fast as you can/are comfortable with and sit down slowly. | 3 min | 4 min |

Please, put in all the information asked in the diary for each session you have performed. Also, note the date for each session. Put in the resistance exercises you have chosen, number of sets you have performed, repetitions in the **last set**, and your perceived dyspnea and muscular fatigue in the **very end** of the **last set**.

***Endurance training***

For the endurance training, please fill in the performed time or the number of performed intervals in the “Endurance” section. Note your perceived dyspnea at the **very end** of the session before cool down.

**Instructions HIIT walk**: Begin the session with a warm-up for 5 minutes at a walking pace corresponding to a Borg dyspnea rating ≤ 3. For the intervals (10 s fast pace walking, 50 s slow pace walking), choose a pace corresponding to 4-8 on the Borg dyspnea scale, and note your perceived Borg dyspnea score after **the very last interval.** End the session with a cool down at the same intensity as the warm-up.

**Instructions MICT walk:** Begin the session with a warm-up for 5 minutes at a walking pace corresponding to a Borg dyspnea rating ≤ 3. Choose a pace (for 20 minutes) that corresponds to 4-6 on the Borg dyspnea scale and note your perceived Borg dyspnea score **at the end of the last minute.** End the session with a cool down on the same intensity as the warm-up.

*IMPORTANT! Do not exercise if you feel sick (for example more coughing, wheezing, breathless or sputum than usual. Need of inhaler/nebulizer more than usual. Less energy, appetite, or sleep than usual).*

***Anything you want to share?***

Please write down anything that you would like to share in relation to the training sessions and/or your health.

### TRAINING DIARY

**Name:** *John Doe*

**Week:** *Week 19 - 20*

**Resistance training** *(an example is given)*

| **Date:** 14/11/2023 | | | **Session nr:** 1 | |
| --- | --- | --- | --- | --- |
| **RT Exercise** | **Sets [max. 2]** | **Reps [goal: 8–12]** | **Dyspnea** | **Heaviness** |
| *Sit to stand level 1* | *2* | *12* | *5* | *6* |
| *Calf raises* | *2* | *12* | *4* | *6* |
| *Chest press* | *2* | *10* | *5* | *5* |
| *Shoulder flexion* | *2* | *10* | *5* | *5* |
| **Date:** | | | **Session nr:** | |
| **RT Exercise** | **Sets [max. 2]** | **Reps [goal: 8–12]** | **Dyspnea** | **Heaviness** |
|  |  |  |  |  |
|  |  |  |  |  |
|  |  |  |  |  |
|  |  |  |  |  |
| **Date:** | | | **Session nr:** | |
| **RT Exercise** | **Sets [max. 2]** | **Reps [goal: 8–12]** | **Dyspnea** | **Heaviness** |
|  |  |  |  |  |
|  |  |  |  |  |
|  |  |  |  |  |
|  |  |  |  |  |
| **Date:** | | | **Session nr:** | |
| **RT Exercise** | **Sets [max. 2]** | **Reps [goal: 8–12]** | **Dyspnea** | **Heaviness** |
|  |  |  |  |  |
|  |  |  |  |  |
|  |  |  |  |  |
|  |  |  |  |  |

**Endurance training** *(an example is given)*

| **Session nr** | **Date** | **Type** | **Time (min)** | **Dyspnea** | **RPE** |
| --- | --- | --- | --- | --- | --- |
| *1* | *16/11/2023* | *MICT walk* | *20 min* | *5* | *14* |
| 2 |  |  |  |  |  |
| 3 |  |  |  |  |  |
| 4 |  |  |  |  |  |

**Anything you want to share?**

## FOLLOW-UP PHONE CALL IN PHASE 2 (USUAL CARE AND INTERVENTION GROUPS)

**Standard questions that will be asked every third month during the follow-up phone call in Phase 2.**

**Exacerbations**

- How many times did you experience symptoms of possible COPD exacerbations, such as increased wheezing, breathlessness and/or coughing, in the last 3 months?
  - *Warning signals of a COPD exacerbation are more wheezy or breathless than normal, more coughing than normal, change in color and amount of sputum, less energy for usual activities, loss of appetite or sleep, need for inhaler or nebulizer more often than normal, signs of fever or first signs of a cold.*
- Did you visit your general practitioner, the emergency room or the hospital in relation to those symptoms? *(Information on any medical assistance is gathered)*
- Did you require a change in medication and/or did you require a hospitalization in relation to those symptoms? *(Information on medication change, and duration for hospital stay is gathered)*

**Medication**

- Have you had any other change in your medication in the last three months?

**Other remarks/events**

- Any other important remarks/events that you think is important to share with the research team? *For example: change in physical activity behavior, starting of exercise training, any hospitalization that is not related to your COPD, surgery, change in health status.*

### **GENERAL TEMPLATE FOLLOW-UP PHONE CALL PHASE 2**

**Name:** *John Doe*

**Period:** *Month 3 to month 6*

**Exacerbations** *(an example is given on the first row; GP = general practitioner)*

| **Date** | **More wheezy or breathless than normal?** | | **More coughing than normal?** | | **Change in amount or color of sputum?** | | **In need of medical assistance?** | | **Change in medication?** | | **Hospitalization necessary?** | |
| --- | --- | --- | --- | --- | --- | --- | --- | --- | --- | --- | --- | --- |
|  | **YES** | **NO** | **YES** | **NO** | **YES** | **NO** | **YES** | **NO** | **YES** | **NO** | **YES** | **NO** |
| *4/10/’23* |  | X | X |  |  | X | GP |  |  | X |  | X |
|  |  |  |  |  |  |  |  |  |  |  |  |  |
|  |  |  |  |  |  |  |  |  |  |  |  |  |
|  |  |  |  |  |  |  |  |  |  |  |  |  |
|  |  |  |  |  |  |  |  |  |  |  |  |  |
|  |  |  |  |  |  |  |  |  |  |  |  |  |
|  |  |  |  |  |  |  |  |  |  |  |  |  |

Other remarks regarding exacerbations *(an example is given):*

*No remarks*

**Medication change** *(write down the date, name of the medication, dose and frequency; an example is given)*

*1/12/2023: Simvastatin Teva – 20 mg – 1 tablet per day*

**Other remarks/events** *(an example is given)*

*Hospitalized for knee-surgery on 06/12/2023 – 3 days hospitalized + ongoing rehabilitation (2x/week) for the next 6 months.*

**Visual feedback Kinomap application**

**Figure S4 and S5**

**
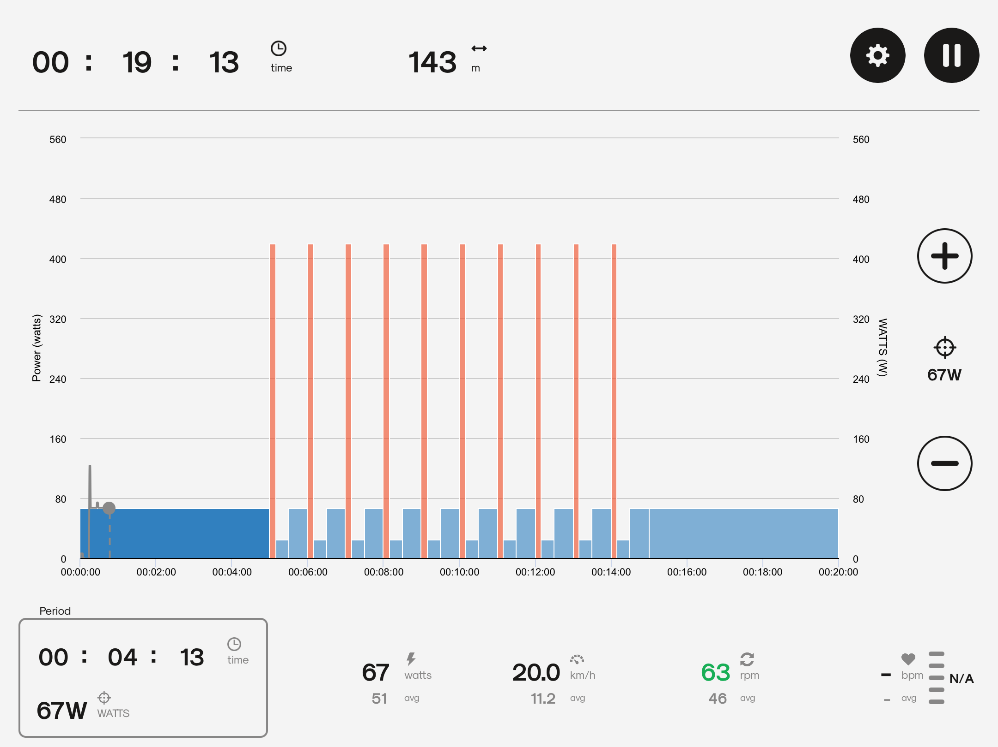
**

**
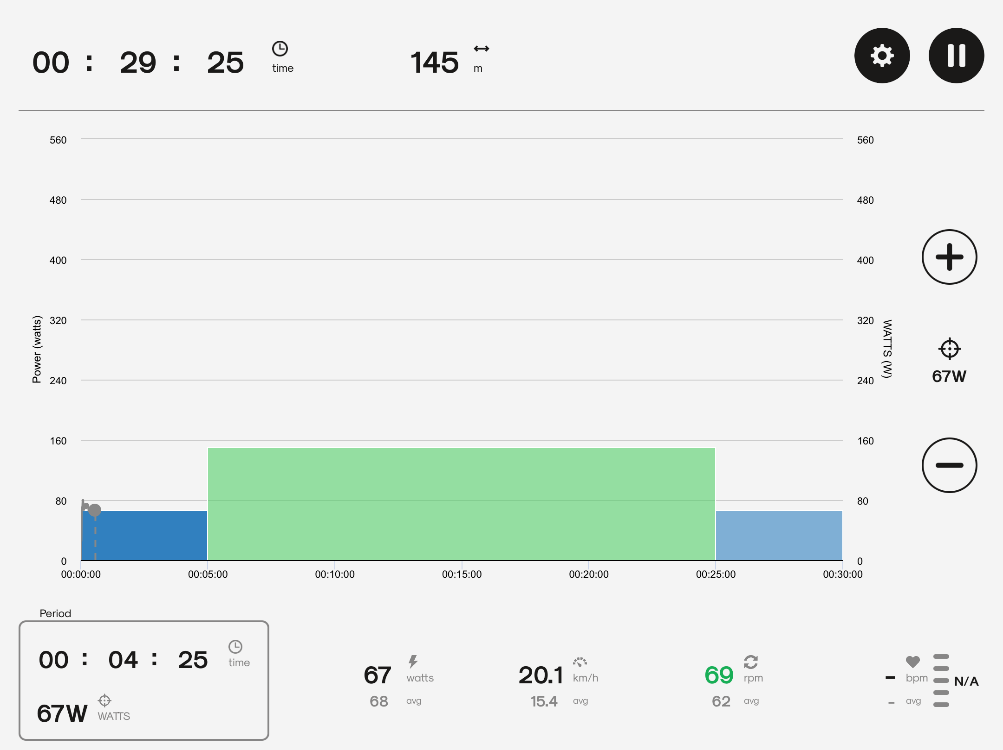
**

**Figure S5:** Visual feedback from the Kinomap application during a MICT session. *With permission.*

**Figure S4:** Visual feedback from the Kinomap application during a supramaximal HIIT session. *With permission.*
