# Supplementary material for: Effects and mechanisms of supramaximal high-intensity interval training on extrapulmonary manifestations in people with and without chronic obstructive pulmonary disease (COPD-HIIT): study protocol for a multi-centre, randomized controlled trial
Source: Trials. 2024 Oct 8;25:664. doi: 10.1186/s13063-024-08481-3 (PMC11460198; doi:10.1186/s13063-024-08481-3)
Supplement: Supplementary file 5 — Additional file 5: Figure S1-S3. [file 13063_2024_8481_MOESM5_ESM.pdf]

Figure S1 - Home exercises - Muscle Strength

• Select 4 exercises each session (one of the sit to stand exercises should always be included). • Do each selected exercise with resistance (weight/level) that allows you to complete the exercise a maximum of 8-12 repetitions/times without pause. If you can't do 8 times, the resistance is too great, and you should reduce the resistance. If you can do more than 12 times, the exercise is too easy and you should increase the resistance/weight or the level.• To increase the loading (weight) on the elastic band exercises, "shorten" the length of the elastic band by moving you grip closer to the origin of the elastic band. E.g. in the shoulder exercise by moving your grip further down on the elastic closer to your feet.
• To increase the difficulty of the sit to stand or the calf raise exercises you change the level.

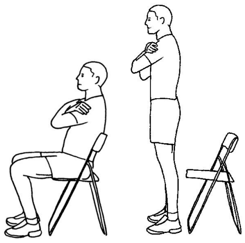
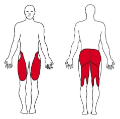

1. Sit to stand - Level 1

Sit on a chair with your arms crossed in front of the chest. Move your torso forward and get up to a standing position. **Sets: 2 , Reps: 8-12 , Pause: 1 min 0 sec**

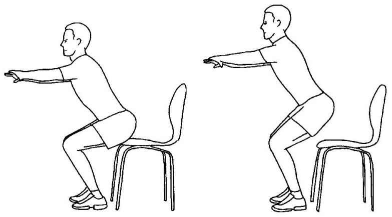
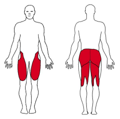

2. Sit to stand - Level 2

Stand in front of a chair. Legs are a hip-width distance apart. Knees and toes are pointing straight forward and arms are kept stretched in front of your body. Do a knee bend, and bend down as far as you can without touching the chair and then stand up again and repeat.
Sets: 2 , Reps: 8-12 , Pause: 1 min 0 sec

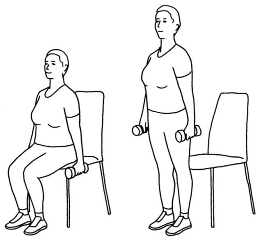
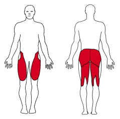

3. Sit to stand - Level 3

Sit on a chair, place an elastic band under your feet and hold it in your hands. The elastic band should be slightly stretched in the start position. Get up to standing position holding the elastic band in your hands becoming more and more stretched as you stand up. Slowly sit down again. Repeat.
Sets: 2 , Reps: 8-12 , Pause: 1 min 0 sec

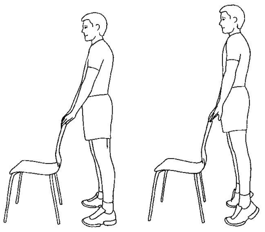
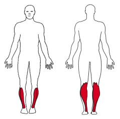

4. Calf Raise - Level 1

Stand up with both hands supported by the back of a chair, a handle or similar. Stand with the feet approx. shoulders width apart. Lift both heels and stand on the toes. Return to the starting position and repeat.
Sets: 2 , Reps: 8-12 , Pause: 1 min 0 sec

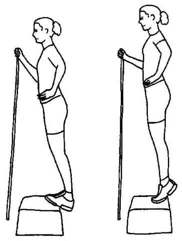
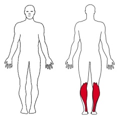

5. Calf Raise - Level 2

Stand on the edge of a step or a bench. The heels should not be touching the step. Your feet should be with approximately a hip-width distance between them. Lift your heels up and press until you are standing on your toes- this movement must be done quickly. Then slowly lower, and return to starting position, and repeat. The exercise can be done with-or without support.
Sets: 2 , Reps: 8-12 , Pause: 1 min 0 sec

Figure S1 - Home exercises - Muscle Strength

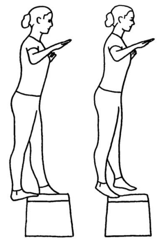
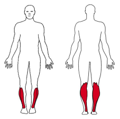

6. Calf Raise - Level 3
Stand single legged on a box with your heel off the edge. Keep your balance and raise your heel. Slowly lower back down and repeat.
Sets: 2 , Reps: 8-12 , Pause: 1 min 0 sec

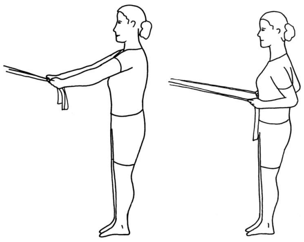
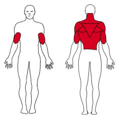

7. Rowing
Fasten the elastic band on a firm object. Stand with your feet apart, facing the wall bar with a handle in each hand. Hold your arms straight in front of you and pull the handles toward your abdomen. Slowly return to the start position and repeat.
Sets: 2 , Reps: 8-12 , Pause: 1 min 0 sec

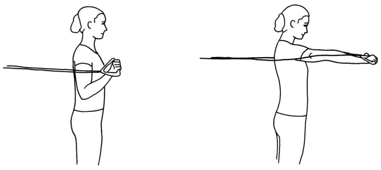
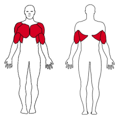

8. Chest Press
Fasten the elastic band in a firm object. Grasp the resistance band with bent elbows and palms facing forwards. Press the resistance band forwards until the elbows are fully extended. Add a shoulder protraction by pressing your arms further forward and pushing your upper back a bit backwards. Slowly return to the starting position. The exercise should be painless and performed with an even movement.
Sets: 2 , Reps: 8-12 , Pause: 1 min 0 sec

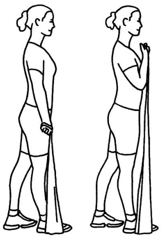
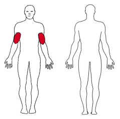

9. Biceps
Place the middle of the elastic band under your feet. Stand with arms straight down along your sides with your thumbs pointing forwards. Bend your elbows while turning thumbs outward. Lower slowly back to the starting position and repeat.
Sets: 2 , Reps: 8-12 , Pause: 1 min 0 sec

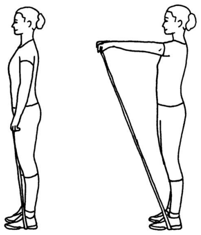
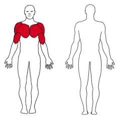

10. Shoulders
Grasp the resistance band with both hands and fasten it beneath your feet by standing on the middle of it. Hold the resistance band in front of your body with palms facing backwards. Raise both hands forwards and up to shoulder level with straight arms. Slowly return to the starting position. The exercise should be painless and performed with an even movement.
Sets: 2 , Reps: 8-12 , Pause: 1 min 0 sec

Figure S2 - Home exercises - Muscle Endurance

• Select 4 exercises each session (one of the sit to stand exercises should always be included). • Do each selected exercise with resistance (weight/level) that allows you to complete the exercise a maximum of 15-25 repetitions/times without pause. If you can't do 15 times, the resistance is too great, and you should reduce the resistance. If you can do more than 25 times, the exercise is too easy and you should increase the resistance/weight or the level.• To increase the loading (weight) on the elastic band exercises, "shorten" the length of the elastic band by moving you grip closer to the origin of the elastic band. E.g. in the shoulder exercise by moving your grip further down on the elastic closer to your feet. • To increase the difficulty of the sit to stand or the calf raise exercises you change the level.

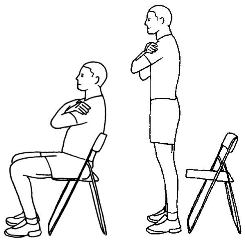
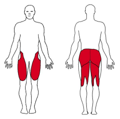

1. Sit to stand - Level 1

Sit on a chair with your arms crossed in front of the chest. Move your torso forward and get up to a standing position. **Sets: 2 , Reps: 15-25 , Pause: 1 min 0 sec**

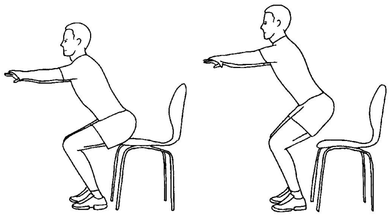
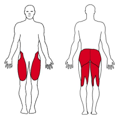

2. Sit to stand - Level 2

Stand in front of a chair. Legs are a hip-width distance apart. Knees and toes are pointing straight forward and arms are kept stretched in front of your body. Do a knee bend, and bend down as far as you can without touching the chair and then stand up again and repeat.
Sets: 2, Reps: 15-25 , Pause: 1 min 0 sec

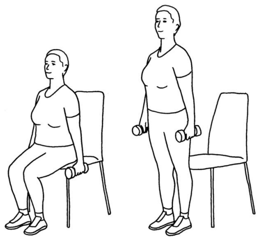
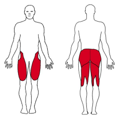

3. Sit to stand - Level 3

Sit on a chair, place an elastic band under your feet and hold it in your hands. The elastic band should be slightly stretched in the start position. Get up to standing position holding the elastic band in your hands becoming more and more stretched as you stand up. Slowly sit down again. Repeat.
Sets: 2 , Reps: 15-25 , Pause: 1 min 0 sec

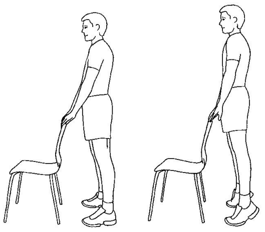
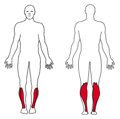

4. Calf Raise - Level 1

Stand up with both hands supported by the back of a chair, a handle or similar. Stand with the feet approx. shoulders width apart. Lift both heels and stand on the toes. Return to the starting position and repeat.
Sets: 2 , Reps: 15-25 , Pause: 1 min 0 sec

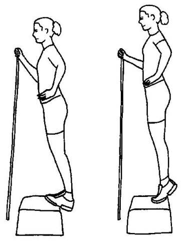
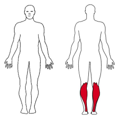

5. Calf Raise - Level 2

Stand on the edge of a step or a bench. The heels should not be touching the step. Your feet should be with approximately a hip-width distance between them. Lift your heels up and press until you are standing on your toes- this movement must be done quickly. Then slowly lower, and return to starting position, and repeat. The exercise can be done with-or without support.
Sets: 2 , Reps: 15-25 , Pause: 1 min 0 sec

Figure S2 - Home exercises - Muscle Endurance

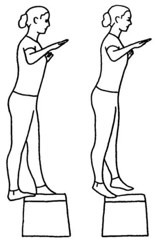
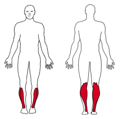

6. Calf Raise - Level 3

Stand single legged on a box with your heel off the edge. Keep your balance and raise your heel. Slowly lower back down and repeat.

Sets: 2 , Reps: 15-25 , Pause: 1 min 0 sec

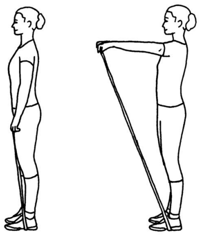
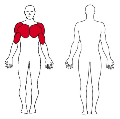

7. Shoulders

Grasp the resistance band with both hands and fasten it beneath your feet by standing on the middle of it. Hold the resistance band in front of your body with palms facing backwards. Raise both hands forwards and up to shoulder level with straight arms. Slowly return to the starting position. The exercise should be painless and performed with an even movement.

Sets: 2 , Reps: 15-25 , Pause: 1 min 0 sec

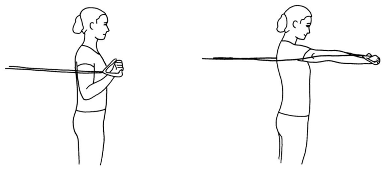
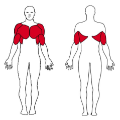

8. Chest Press

Fasten the elastic band in a firm object. Grasp the resistance band with bent elbows and palms facing forwards. Press the resistance band forwards until the elbows are fully extended. Add a shoulder protraction by pressing your arms further forward and pushing your upper back a bit backwards. Slowly return to the starting position. The exercise should be painless and performed with an even movement.

Sets: 3 , Reps: 15-25 , Pause: 1 min 0 sec

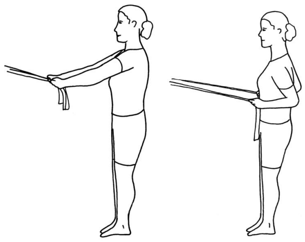
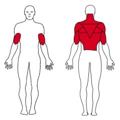

9. Rowing

Fasten the elastic band on a firm object. Stand with your feet apart, facing the wall bar with a handle in each hand. Hold your arms straight in front of you and pull the handles toward your abdomen. Slowly return to the start position and repeat.

Sets: 2 , Reps: 15-25 , Pause: 1 min 0 sec

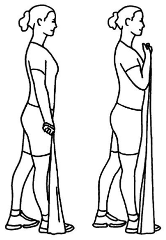
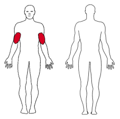

10. Biceps

Place the middle of the elastic band under your feet. Stand with arms straight down along your sides with your thumbs pointing forwards. Bend your elbows while turning thumbs outward. Lower slowly back to the starting position and repeat.

Sets: 2 , Reps: 15-25 , Pause: 1 min 0 sec

Figure S3 - Home exercises - Muscle Power

• Select 3 exercises each session (one of the sit to stand exercises should always be included). • Do each selected exercise with resistance (weight/level) that you use for the "muscle endurance" training, i.e., that you can do 15-25 repetitions on. However, only perform 8-12 repetitions but perform the exercise AS FAST AS YOU CAN/ARE COMFORTABLE WITH in the first phase of the movement, and then slow in the second phase. See details in each exercise. • Increase/change in loading follows the changes made in the "muscle endurance" program, e.g., if you in the "muscle endurance" program are on level 2 in the sit to stand exercise, you should also select Sit to stand - Level 2 in the "muscle power" training.

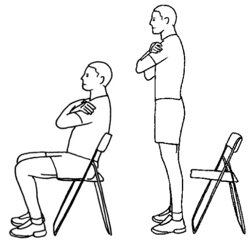
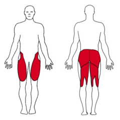

1. Sit to stand - Level 1

Sit on a chair with your arms crossed in front of the chest. Move your torso forward and get up to a standing position **as fast as you can.** Return slowly to a sitting position, and repeat.

Sets: 2 , Reps: 8-12 , Pause: 3 min 0 sec

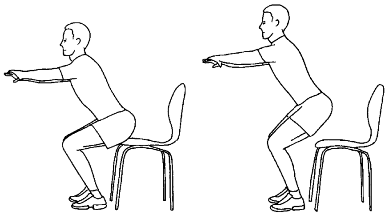
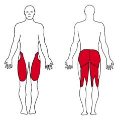

2. Sit to stand - Level 2

Stand in front of a chair. Legs are a hip-width distance apart. Knees and toes are pointing straight forward and arms are kept stretched in front of your body. Do a knee bend, and bend down as far as you can without touching the chair and then stand up again **as fast as you can**. Return slowly to a sitting position, and repeat.

Sets: 2 , Reps: 8-12 , Pause: 3 min 0 sec

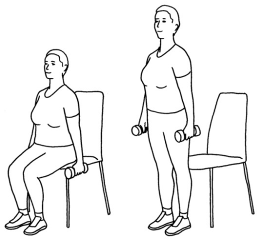
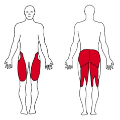

3. Sit to stand - Level 3

Sit on a chair, place an elastic band under your feet and hold it in your hands. The elastic band should be slightly stretched in the start position. Get up to standing position **as fast as you can** holding the elastic band in your hands becoming more and more stretched as you stand up. Slowly sit down again. Repeat.

Sets: 2 , Reps: 8-12 , Pause: 3 min 0 sec

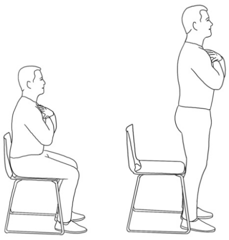
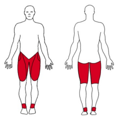

4. Sit to stand - Level 4 (with jump)

Sit in the middle of the chair. Place your arms crossed over your chest. Keep your feet flat on the floor. **Raise up as fast as possible and if possible make a small jump**. Therafrer sit down slowly, and repeat.

Sets: 2 , Reps: 8-12 , Pause: 3 min 0 sec

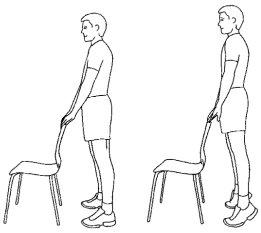
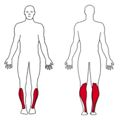

5. Calf Raise - Level 1

Stand up with both hands supported by the back of a chair, a handle or similar. Stand with the feet approx. shoulders width apart. Lift both heels **as fast as you can** and stand on the toes. Return to the starting position and repeat.

Sets: 2 , Reps: 8-12 , Pause: 3 min 0 sec

Figure S3 - Home exercises - Muscle Power

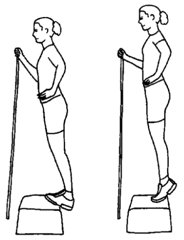

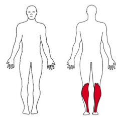

6. Calf Raise - Level 2

Stand on the edge of a step or a bench. The heels should not be touching the step. Your feet should be with approximately a hip-width distance between them. Lift your heels up **as fast as you can** and press until you are standing on your toes- **this movement must be done quickly.** Then slowly lower, and return to starting position, and repeat.

Sets: 2 , Reps: 8-12 , Pause: 3 min 0 sec

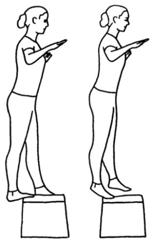

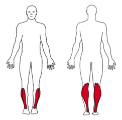

7. Calf Raise - Level 3

Stand single legged on a box with your heel off the edge. Lift your heels up **as fast as you can** and press until you are standing on your toes- **this movement must be done quickly.** Then slowly lower, and return to starting position, and repeat. The exercise can be done with-or without support.

Sets: 2 , Reps: 8-12 , Pause: 3 min 0 sec

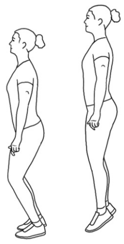

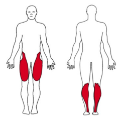

8. Calf Raise - Level 4 (with jump)

Stand with the feet approx. shoulders width apart. Ben you knees sligtly and then extend you legs ast as you can **and make a small jump** Return to the starting position and repeat.

Sets: 2 , Reps: 8-12 , Pause: 3 min 0 sec

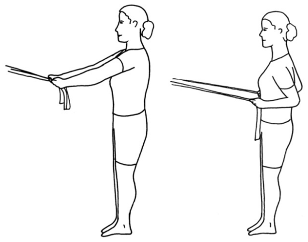

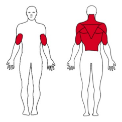

9. Rowing

Fasten the elastic band on a firm object. Stand with your feet apart, facing the wall bar with a handle in each hand. Hold your arms straight in front of you and pull the handles toward your abdomen **as fast as you can.** Slowly return to the start position and repeat.

Sets: 2 , Reps: 8-12 , Pause: 3 min 0 sec

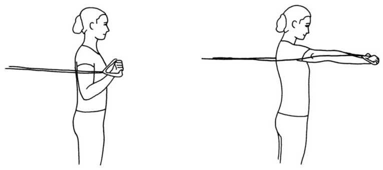

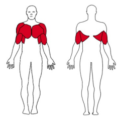

10. Chest Press

Fasten the elastic band in a firm object. Grasp the resistance band with bent elbows and palms facing forwards. Press the resistance band forwards until the elbows are fully extended **as fast as you can**. Add a shoulder protraction by pressing your arms further forward and pushing your upper back a bit backwards. Slowly return to the starting position. The exercise should be painless and performed with an even movement.

Sets: 2 , Reps: 8-12 , Pause: 3 min 0 sec
