# Supplementary material for: Effects and mechanisms of supramaximal high-intensity interval training on extrapulmonary manifestations in people with and without chronic obstructive pulmonary disease (COPD-HIIT): study protocol for a multi-centre, randomized controlled trial
Source: Trials. 2024 Oct 8;25:664. doi: 10.1186/s13063-024-08481-3 (PMC11460198; doi:10.1186/s13063-024-08481-3)
Supplement: Supplementary file 6 — Additional file 6: SPIRIT Item 2b WHO Trial Data Set. [file 13063_2024_8481_MOESM6_ESM.docx]

**World Health Organization Trial Registration Data Set**

| **Primary registry and trial identifying number** | Clinicaltrials.gov, NCT06068322 |
| --- | --- |
| **Date of registration in primary registry** | 2023-09-28 |
| **Secondary identifying numbers** | Swedish Ethical Review Authority (#2023-00747-01 and 2023-00747-02), Committee for Medical Ethics of Hasselt University (Hasselt, Belgium) (#B3712023000012). |
| **Sources of monetary or material support** | The Swedish Research Council (Grant number: 2020-01296), The Swedish Heart and Lung Foundation (Grant number: 20210146, 20230341), The European Research Council (Grant number: 101078602), Strategic Research Area – Health Care Science and Special Research Fund Hasselt University (Grant number: BOF23DOC49). |
| **Primary sponsor** | Umeå University |
| **Secondary sponsor** | N/A |
| **Contact for public queries** | Assoc. Prof. Andre Nyberg, Principal Investigator  andre.nyberg@umu.se, +46 90 786 66 39  Section of Physiotherapy, Department of Community Medicine and Rehabilitation, Umeå University, Johan Bures väg 12, 901 87 Umeå |
| **Contact for scientific queries** | Assoc. Prof. Andre Nyberg, Principal Investigator  andre.nyberg@umu.se, +46 90 786 66 39  Section of Physiotherapy, Department of Community Medicine and Rehabilitation, Umeå University, Johan Bures väg 12, 901 87 Umeå |
| **Public title** | Effects of high-intensity interval training in chronic obstructive pulmonary disease |
| **Scientific title** | Effects and mechanisms of supramaximal High-Intensity Interval Training on extrapulmonary manifestations in people with and without Chronic Obstructive Pulmonary Disease (COPD-HIIT) |
| **Countries of recruitment** | Sweden, Belgium |
| **Health conditions or problems studied** | Chronic obstructive pulmonary disease and healthy human volunteers |
| **Interventions** | Experimental arm: Supramaximal high-intensity interval training |
|  | Active comparator: Moderate-intensity continuous training |
|  | Passive comparator: Usual care (Chronic obstructive pulmonary disease) |
| **Key inclusion and exclusion criteria** | Eligible age: 60 years and older  Eligible sex: Male or female |
|  | Inclusion criteria:  Independent in activities of daily living  For people with COPD: Spirometry confirmed COPD diagnosis (FEV1/FVC < 0.70)  Healthy controls: Normal lung function |
|  | Exclusion criteria:  Conditions and diseases that are unstable and/or prohibits exercise or tests, based on screening by a physician.  Other lung conditions.  Medical conditions and treatments with known effects on brain function and cognition |
| **Study type** | Interventional |
|  | Allocation: Randomized intervention |
|  | Masking: Assessors and data analyst |
|  | Assignment: Parallel |
|  | Purpose: Treatment (improve outcomes) |
| **Date of first enrolment:** | 2023-11-09 |
| **Target sample size** | 92 with COPD (active intervention), 46 with COPD (usual care), 70 healthy controls (active interventions) |
| **Recruitment status** | Recruiting |
| **Primary outcomes** | Change from baseline cardiorespiratory fitness (ml O2/min/kg), global cognitive function (Z-score) and quadriceps power (watt) at 3 months and 24 months. |
| **Key secondary outcomes** | Change from baseline exercise tolerance (duration at constant work-load test), brain structure (MRI), brain function (fMRI), neuroinflammation (PET/CT), systemic inflammation (blood sample), neurotrophic factors (blood sample), health-related quality of life (EQ-5D-5L) and vastus lateralis morphology, enzyme activity and mitochondrial biogenesis. |
