# Supplementary material for: Effects and mechanisms of supramaximal high-intensity interval training on extrapulmonary manifestations in people with and without chronic obstructive pulmonary disease (COPD-HIIT): study protocol for a multi-centre, randomized controlled trial
Source: Trials. 2024 Oct 8;25:664. doi: 10.1186/s13063-024-08481-3 (PMC11460198; doi:10.1186/s13063-024-08481-3)
Supplement: Supplementary file 7 — Additional file 7: Model consent form COPD English. [file 13063_2024_8481_MOESM7_ESM.docx]

**Information for research participants**

We would like to ask you if you would like to participate in a research project. In this document, you will find information about the project and what it means to participate.

**What is this project and why do you want me to participate?**

Physical exercise in the form of endurance training can have positive effects on, among other things, physical performance and brain functions, as well as bring great health benefits. This is especially true for older individuals and people with chronic diseases. Endurance training can be done at different intensities and workouts can have different lengths. During a workout, the intensity can also be varied in many different ways. One way to control the intensity during exercise is to decide on the person's individual ability what load to train on. By individually dosing and controlling the intensity in this way, there are good conditions for the training to take place at a reasonably high level so that it has the best possible effect while the training is not associated with discomfort or risks.

The purpose of this research study is therefore to investigate the effects and experiences of exercise training on a bicycle carried out in two different ways in people with chronic obstructive pulmonary disease (COPD) and elderly people without COPD, who are not active in exercise. The two programs we examine have some similarities in terms of e.g. how the intensity is controlled, but also differences in how the training is dosed during the workouts.

The research principal/sponsor for the project is Umeå University. The research principal refers to the organisation responsible for the study.

**How is the study conducted?**

Participating in the study means that you first complete a training period of 3 months. You will be randomly assigned to complete one of two different types of cycling training. The draw takes place after you have completed a number of tests before the start of the training period. The study is about comparing the results between these two forms of exercise. The training is carried out on exercise bikes in a training room, two to three times per week, in a smaller group (maximum 8-10 participants). People with COPD and elderly people without COPD may complete the training together. After the first 3 months, you who have COPD will have the opportunity to continue training for 21 months where you will be given exercises to do at home with an offer of continued leader-led training throughout the follow-up period. Whether you want to train at home or take advantage of the offer of continued leader-led training in a group is voluntary, and you can change how you want to do it at any time during the study. Regardless of how you choose to continue, during the follow-up period, we will contact you once every two weeks over the phone to follow up on how the training is going and how we can adapt it to your needs. Every three months, the conversation also includes questions about the impact of the disease on everyday life, quality of life and symptoms.

In addition to the training period, all participants undergo a number of separate test sessions before and after the training periods. All in all, information and measurements will mean 12 occasions for you. Four occasions before the start of the training period, four occasions after the initial 3-month training period and four occasions after 24 months. You do the tests regardless of which group you are assigned to and are estimated to take about 1-3 hours per occasion including rest between the tests. However, the muscle sample described below is only done before the training period and after the initial 3 months (i.e. two muscle biopsies are performed within the study).

The tests that you will perform will include measurements of lung function, fitness, different measures of muscle strength and muscle function, different cognitive abilities (e.g. memory) and two muscle biopsies (tissue samples). When carrying out fitness tests (3), you will also wear a device that, among other things, measures oxygen uptake and respiratory rate during the tests. We will also use a finger prick to take a drop of blood every two to five minutes during the fitness tests. We will also collect blood samples (about 25-30 ml) via a vein in the crook of the arm to investigate inflammatory factors. You will also have an MRI scan (approx. 1 hour and 15 minutes). The purpose of the study is to study the possible effects of the training on the structure and function of the brain. When you are examined in the MRI scanner, you lie still on a couch. The examination is painless and harmless, but some people may experience some discomfort during the examination due to the small space in the device and the noise emitted by the device. To minimize the discomfort of the noise, effective hearing protection is used. Magnetic resonance imaging (MRI) is routinely performed in healthcare. In addition to the review of the images from the MRI scan that is done to answer our research question, the images are also reviewed by a radiologist so as not to miss any abnormal findings that are sometimes seen even in otherwise healthy people. If, in that review, findings emerge that need follow-up, further investigation or treatment, the doctor who receives the referral response from MRI will ensure that this further treatment is carried out.

Then you will be in a PET/CT camera (about 1 hour). In connection with PET/CT scans, a small dose of a radioactive tracer is injected intravenously. The studies included in the study produce about as little radiation as 2-3 years of background radiation. The PET/CT sessions are done at rest, i.e. You don't have to do anything active, but are encouraged to lie still. These examinations provide information about inflammatory processes in the brain.

Before the training period, you will also be asked to wear an physical activity monitor, for one week.

After the training period, all examinations will be repeated during two weeks and you will also be invited to an interview to share your experiences of the training. You will receive a clear schedule where all examination occasions are specified. The total time for participation is 24 months, of which 3 months are leader-led training in groups. After the first 3 months, you who have COPD will have the opportunity to continue training for 21 months where you will be given exercises to do at home with an offer of supervised training throughout the follow-up period. Whether you want to train at home or take advantage of the offer of continued supervised training in a group is voluntary, and you can change how you want to do it at any time during the study.

Regarding the muscle sample, it will be taken into the vastus lateralis muscle (the muscle located on the outside of the thigh) and is done as follows: Local anesthetic is injected with a needle under the skin on the outside of the thigh. After a few minutes, an incision (about 1.5-2 cm) can be made in the skin. A small piece of the muscle, about 2x2x2 mm, is cut loose. It cannot be ruled out that you will feel a rapid transient discomfort or pain during the sampling itself. The incision is closed with a few stitches. Some residual soreness may occur at the incision for 1-2 days after the biopsy. There is a risk that the stitches will not hold the incision together and bleeding will occur. Time for the whole procedure including preparation time is 1 hour. The second muscle sample will be taken using the same procedure in the vicinity of the first muscle sample but not in the exact same place. No muscle sample is taken after 24 months.

After completing the training, interviews are conducted in groups, this is to capture your experiences of completing the training. The interview is expected to take about 1-1.5 hours. The interviews will be based on a question guide with question areas about experience of participating, positive or negative influence, risks or discomfort, practical feasibility and experience of training in a group.

**Possible consequences and risks of participating in the study**

As with other muscle exertion, exercise and tests may cause some muscle soreness. This is not dangerous, but completely normal and the muscle soreness disappears after a few days. Furthermore, a feeling of discomfort can sometimes be experienced during muscle sampling. After muscle sampling, soreness from pressure can sometimes persist for a couple of days after the measurements have ended. There may be short-term burning and pain in connection with blood sampling. Afterwards, there may be a "bruise" at the puncture site.

The training intensity is constantly adapted to your individual ability and your own experience and everyone must be able to complete the training. A transient physical fatigue occurs during the implementation of the training as well as during some of the tests. In previous studies, we have assessed that both the training and the examinations are safe.

In connection with the PET/CT scans, you will receive an intravenous line of access to the arm via which a dose of a radioactive tracer is injected during each examination. The dose you receive is small and does not produce pharmacological effects. During the examination, the head is fixed to the couch with a face mask, which can feel trapped. Magnetic resonance imaging (MRI) can be experienced as noisy and it can also feel trapped. We are in continuous contact with you during the examinations and increase the comfort with, for example, pillows. All brain imaging sessions are performed by experienced nurses employed by Region Västerbotten. Furthermore, the cognitive tests can be perceived as difficult, which can be frustrating. However, they are designed to be difficult and maximum points should be extremely difficult to obtain. Some of the questions we ask are personal (e.g. diseases, medications, lifestyle, education). Images from the MRI scan are examined by a radiologist and there is a risk of abnormalities (secondary findings).

**What happens to my data?**

The project will collect and record information about you.

The purpose of processing your personal data is to provide a more detailed picture of how this type of physical exercise affects people of different ages and genders, for example. The information we intend to collect and process is name, age, address, height, body weight, smoking habits, education, any diagnoses or medications that affect physical work ability, physical activity level and answers to questionnaires regarding mental well-being, quality of life and cognition. You decide if you want to provide us with any information.

Personal data is handled in accordance with the EU's General Data Protection Regulation (2016/679), GDPR. The data will only be processed within the research group. The legal basis for the processing according to Article 6.1. of the EU's General Data Protection Regulation is the public interest for the purpose of research. Your answers and your results will be treated confidentially, and no unauthorized person will be able to access them. The responses will be compiled in de-identified form, and presented in such a way that individual people's responses cannot be traced. Your personal data will be processed throughout the project, which will last for 5 years. Thereafter, the personal data that is required by law to be archived will be archived in accordance with applicable legislation and the University's records management plans, for some data this means at least 10 years. This processing of personal data takes place on the legal basis that the processing is a legal obligation under the EU General Data Protection Regulation, Article 6.1.c.

According to the EU's General Data Protection Regulation and national supplementary legislation, you have the right to: request access to your personal data (request a register extract), have your personal data corrected, have your personal data deleted, have the processing of your personal data restricted.

In certain circumstances, the General Data Protection Regulation and supplementary national legislation allow for exceptions to these rights. For example, the right of access to one's data may be limited by confidentiality requirements, and the right to have data deleted may be limited by archiving rules. Umeå University is the data controller for your personal data in this project. Contact information for Umeå University's Data Protection Officer: E-mail: [pulo@umu.se](mailto:pulo@umu.se). If you are dissatisfied with how your personal data is processed, you have the right to lodge a complaint with the Swedish Authority for Privacy Protection, which is the supervisory authority.

Umeå University is subject to the principle of public access to official documents, which means that all individuals have the right to obtain copies of public documents. Your personal data may therefore also be disclosed in connection with a request for disclosure of public documents, unless the information is covered by confidentiality

**What happens to my samples?**

The samples taken in the study are stored coded in Biobank Norr. Regarding the muscle sampling, your samples will be numbered in chronological order and will be stored in a locked and alarmed freezer room at the Department of Integrative Medical Biology, Umeå University. As with the other test results, all analysis data regarding the muscle samples is confidential and will be coded so that no one can trace the results of your muscle samples to your name or social security number. The principal (responsible) for the biobank is Region Västerbotten

You have the right to say no to the samples being saved. If you consent to the samples being saved, you have the right to later withdraw (withdraw) that consent. In that case, your samples will be discarded or de-identified. If you wish to withdraw your consent, please contact the researcher responsible for the study, contact information can be found at the bottom of the page.

The samples may only be used in the manner to which you have given your consent. If there is additional research that has not yet been planned, the Ethical Review Board will decide whether you should be asked again.

**How do I get information about the results of the study?**

According to the EU's General Data Protection Regulation, you have the right to access the data about you that is processed in the study free of charge and, if necessary, have any errors corrected. You can also request that information about you be deleted and that the processing of your personal data be restricted. If you want to access the information, you should contact the responsible researcher for the study, contact information can be found at the bottom. You do not need to take part in any analysis results if you do not want to.

**Insurance and compensation**

As a research subject, you are covered by the Legal, Financial and Administrative Services Agency's insurance for all tests performed at Umeå University and the Unit of Physiotherapy. Muscle biopsies and exercise will be performed at the University Hospital of Umeå, Sweden and you will then be covered by the patient insurance. All tests and training are free of charge. On completion of the study, the one financial compensation if 2700 SEK whereof 1800 taxable and 900 tax-free (blood/tissue). Partial payment is made in the event of discontinuation of the study.

**Participation is voluntary.**

Your participation is voluntary, and you can choose to cancel your participation at any time. If you choose not to participate or wish to cancel your participation, you do not need to state why, nor will it affect your future care or treatment. If you wish to cancel your participation, please contact the person responsible for the study (see below).

**Responsible for the study**

Responsible for the study is Andre Nyberg. Associate professor, Licensed Physiotherapist, Email: andre.nyberg@umu.se, Phone: 090 786 6639. Available 08:00-16:45. Department Department of Physiotherapy, Department of Community Medicine and Rehabilitation, Umeå University, 901 87 Umeå.

**Consent to participate in the study**

I have received oral and written information about the study and have had the opportunity to ask questions. I am allowed to keep the written information.

I agree to participate in the study High-intensity interval training adapted for people with COPD or without COPD .

I agree that my samples will be stored in a biobank in the manner described in the research subject information.

| Place and date | Signature |
| --- | --- |
|  |  |
